# Evaluation of functionalized graphene oxide as a nanostructured sensor for lead ion detection in aqueous solutions via quartz crystal microbalance

**DOI:** 10.1038/s41598-026-50889-1

**Published:** 2026-05-11

**Authors:** Haitham A. Kilany, Rabab M. Elsherif, Soha A. Abdel Gawad

**Affiliations:** 1 Faculty of Postgraduate Studies for Nanotechnology, Cairo University, Giza, Egypt; 2 Faculty of Science, Cairo University, Giza, 12613 Egypt

**Keywords:** Graphene oxide, 3-mercaptopropyltrimethoxysilane, Quartz crystal microbalance, Lead ion, Sensor selectivity, Water treatment, Chemistry, Environmental sciences, Materials science, Nanoscience and technology

## Abstract

Since heavy metals are not subject to biodegradation and can accumulate in living organisms, rising levels of lead ion (Pb^2+^) and other heavy metal pollutants in water sources pose a significant threat to environmental health and public safety. Although conventional detection techniques are accurate, they are often expensive, time-consuming, and unsuitable for field use. Accordingly, it is highly required but remains exceedingly challenging to improve a facile detection approach for heavy metal pollutants in water. Here, we present the creation of a novel, cost-effective, and highly sensitive quartz crystal microbalance (QCM) sensor designed for the detection of Pb^2+^, utilizing 3-mercaptopropyltrimethoxysilane-functionalized graphene oxide (3-MPTMS-GO). A modified Hummer’s method was employed to synthesize GO, which was then functionalized with 3-MPTMS through a cross-linking method. Various characterization techniques, including Transmission electron microscopy (TEM), Atomic force microscopy (AFM), X-ray diffraction (XRD), Fourier transform infrared spectroscopy (FT-IR), X-ray Photoelectron Spectroscopy (XPS), Raman spectroscopy, UV-Vis spectroscopy, contact angle measurements, and Zeta potential analysis, were performed to confirm effective functionalization and enhanced surface properties. The QCM sensor coated with 3-MPTMS-GO demonstrated excellent sensitivity to Pb^2+^ across various pH levels at different concentrations: 0.05 mg L^− 1^, 1 mg L^− 1^, and 2 mg L^− 1^. Through the calibration curve, the sensor revealed a linear detection range of Pb^2+^ concentration from 0.05 mg L^− 1^ to 1.5 mg L^− 1^ (R^2^ = 0.999) with a limit of detection (LOD) of 0.01 mg L^− 1^, which aligns with WHO standards. Furthermore, the sensor verified outstanding regeneration capability over 10 reuse cycles using EDTA treatment and excellent stability towards selective lead ions. This preliminary investigation illustrates the significant potential of the 3-MPTMS-GO-based QCM sensor as a proof-of-concept platform for Pb^2+^ detection under controlled laboratory conditions, necessitating extensive validation prior to implementation in practical environmental monitoring applications.

**Supplementary Information:**

The online version contains supplementary material available at 10.1038/s41598-026-50889-1.

## Introduction

The swift urban growth and industrial expansion in developing countries are worsening global water scarcity. The pollution of water by heavy metal ions has recently surged to concerning levels, posing threats to the environment and ecosystem^1^. Industrial wastewater treatment exposes us to dangerous heavy metal ions like mercury (Hg^2+^), cadmium (Cd^2+^), chromium (Cr^3+^), lead (Pb^2+^), and arsenic (As^3+^). These heavy metal ions do not decompose and build up in living organisms, leading to long-term poisoning^2^. Lead exists in several oxidation states, including metallic lead Pb⁰, Pb⁺, and Pb²⁺ however, Pb²⁺ is the most stable and environmentally relevant form in aqueous systems. Metallic Pb⁰ released from industrial pipelines, batteries, pigments, and wastewater sources is rapidly oxidized to Pb²⁺ in the presence of dissolved oxygen, making Pb²⁺ the predominant soluble and mobile species responsible for lead toxicity in water. Pb²⁺ is of particular concern because studies indicate that exposure to Pb^2+^ in the workplace and environment may be associated with different disorders affecting the nervous system, blood formation, liver, and kidney functions. The main routes people are exposed to lead are through food and water that have been contaminated^3^. According to the World Health Organization (WHO), the maximum allowable limit for Pb^2+^ in drinking water at 0.01 mg L^− 1^^4^. Therefore, it is highly predictable to detect Pb^2+^ in an aqueous medium to meet WHO values. To address this challenge, many traditional analytical approaches for Pb^2+^ detection have been utilized, such as atomic absorption spectroscopy, inductively coupled plasma-mass spectroscopy, and electrochemical methods^5^. Although these methods offer remarkable selectivity and sensitivity, they come at a high cost, lack portability, are time-consuming, and frequently necessitate multiple procedures and skilled personnel. Furthermore, they demand significant labor^6^. Considering the limitations of these methods, it is highly advantageous to have approaches that are low-cost, uncomplicated, rapid, and portable. Recently, quartz crystal microbalance (QCM) sensors have emerged as a promising option for detecting analytes, offering numerous benefits such as high sensitivity, cost-effectiveness, simple installation, and the ability to monitor analytes in real time and directly on-site. QCM serves as a high-resolution mass transducer for biological and chemical sensors, as it detects fluctuations in the resonance frequency of quartz crystal surfaces in real time^7–11^. It is commonly utilized in environmental testing, biology, life sciences, analytical chemistry, and pharmaceutical sciences^12–14^. Enhancing the surface characteristics of sensing materials on QCM sensors, as advancements in nanomaterials fabrication occur, aims to provide swift responses, significant sensitivity, and strong selectivity for the detection of metal ions^15–17^. Among nanomaterials, GO as a form of carbon material has been explored for various applications due to its exceptional characteristics^18–22^. The functionalization of GO is defined as GO that has been subjected to additional chemical modifications by bonding specific functional groups or molecules to its surface through chemical methods. The functionalization offers several advantages, such as increased dispersibility, customizable surface chemistry, enhanced stability, and improved electrical, optical, and mechanical characteristics^23–26^. Based on this approach, different organosilane functional groups, including 3-aminopropyltriethoxysilane and 3-mercaptopropyltrimethoxysilane (3-MPTMS), have been employed for the functionalization of GO, which enhances its usability^27–29^. Our study aims to develop a more facile QCM sensor that utilizes 3-MPTMS-GO to detect heavy metals, particularly focusing on Pb^2+^. The selection of 3-MPTMS is based on Pearson’s Hard-Soft Acid-Base (HSAB) theory, where lead ions (Pb²⁺) are classified as soft acids with particularly strong affinity for sulfur-containing ligands (soft bases). Literature reports demonstrate that thiol-Pb²⁺ binding constants are typically 10² times higher than those for other divalent cations, providing the theoretical foundation for superior selectivity^30–32^. Moreover, silane coupling agents guarantee stable grafting onto GO that could contribute to surface area enhancement, increase hydrophilicity, and improve the structural robustness of the sensing film deposited on the QCM crystal against degradation during repeated usage. In response to this, we investigated the synthesis, characterization, and application of 3-MPTMS-GO composite as an innovative sensor for QCM uses. The creation of 3-MPTMS-GO involved the silanization of GO with 3-MPTMS. The resultant sensor was characterized through TEM, FT-IR, AFM, XRD, Raman spectroscopy, and Zeta-potential assessments. The QCM sensor based on 3-MPTMS-GO successfully identified Pb^2+^, offering superiorities such as high selectivity, fast real-time response, a low detection limit, good performance across a wide pH spectrum, and excellent stability. The novelty of this study lies in introducing to the best of our knowledge, the first reported application of a QCM sensor based on 3-MPTMS-GO for the detection of Pb²⁺ in aqueous environments and addressing research gaps in existing methods by filling the need for rapid environmental monitoring, offering systematic pH-dependent mechanistic investigation, and overcoming low selectivity, tackling the gap in cost efficiency, and maintaining effective reusability. Overall, the QCM sensor based on 3-MPTMS-GO represents an innovative approach for the detection of Pb²⁺ in aqueous environments, marking a substantial breakthrough in real-time water-quality monitoring applications.

## Materials and methods

### Materials

Graphite powder was provided by Asbury Carbons, USA. Lead acetate (Pb (CH_3_COO)_2_) and potassium permanganate (KMnO_4_) were obtained from Merck. Hydrogen peroxide (H_2_O_2_, 35%), hydrochloric acid (HCl, 37%), sulfuric acid (H_2_SO_4_, 98%), hypophosphoric acid (H_3_PO_4_, 85%), and 3-mercaptopropyltrimethoxysilane (3-MPTMS) were acquired from Fisher. Copper nitrate (Cu (NO_3_)_2_.3H_2_O) and calcium hydroxide (Ca (OH)_2_) were obtained from Alpha Chemika. Chromium nitrate (Cr (NO_3_)_3_.9H_2_O) was acquired from Merck. Sodium hydroxide (NaOH) was sourced from Advent Chembion. Deionized water was utilized for preparing solutions and for cleaning purposes in this study.

### Synthesis of GO

GO was synthesized using a modified Hummer’s method^33,34^. Generally, 5 g of graphite powder was mixed with a solution of H_2_SO_4_ and H_3_PO_4_ in a 9:1 mL ratio. KMnO_4_ (30 g) was then carefully added to the mixture while it was kept in an ice bath. The mixture was stirred until it achieved a dark green color and continued to be stirred at 50 °C for a period of 3 days. The mixture of graphite and acids transformed into a light brown color. Gradually, this graphite mixture was added to an ice-cold solution containing 666 mL of deionized water and 16 mL of 30% H_2_O_2_. This process resulted in a vibrant, golden GO solution. The resulting GO solution was filtered and treated with a 10 wt% HCl solution four times, followed by multiple washes with water until the pH was nearly in the (6.9–7.1) range to form stable oxygenated functional groups valuable for a uniform layer of GO on the quartz crystal, enhancing QCM stability and sensor response. The resulting precipitate was dried in an oven at 90 °C for 24 h, resulting in the production of GO.

### Synthesis of 3-MPTMS-GO

**Scheme 1:**
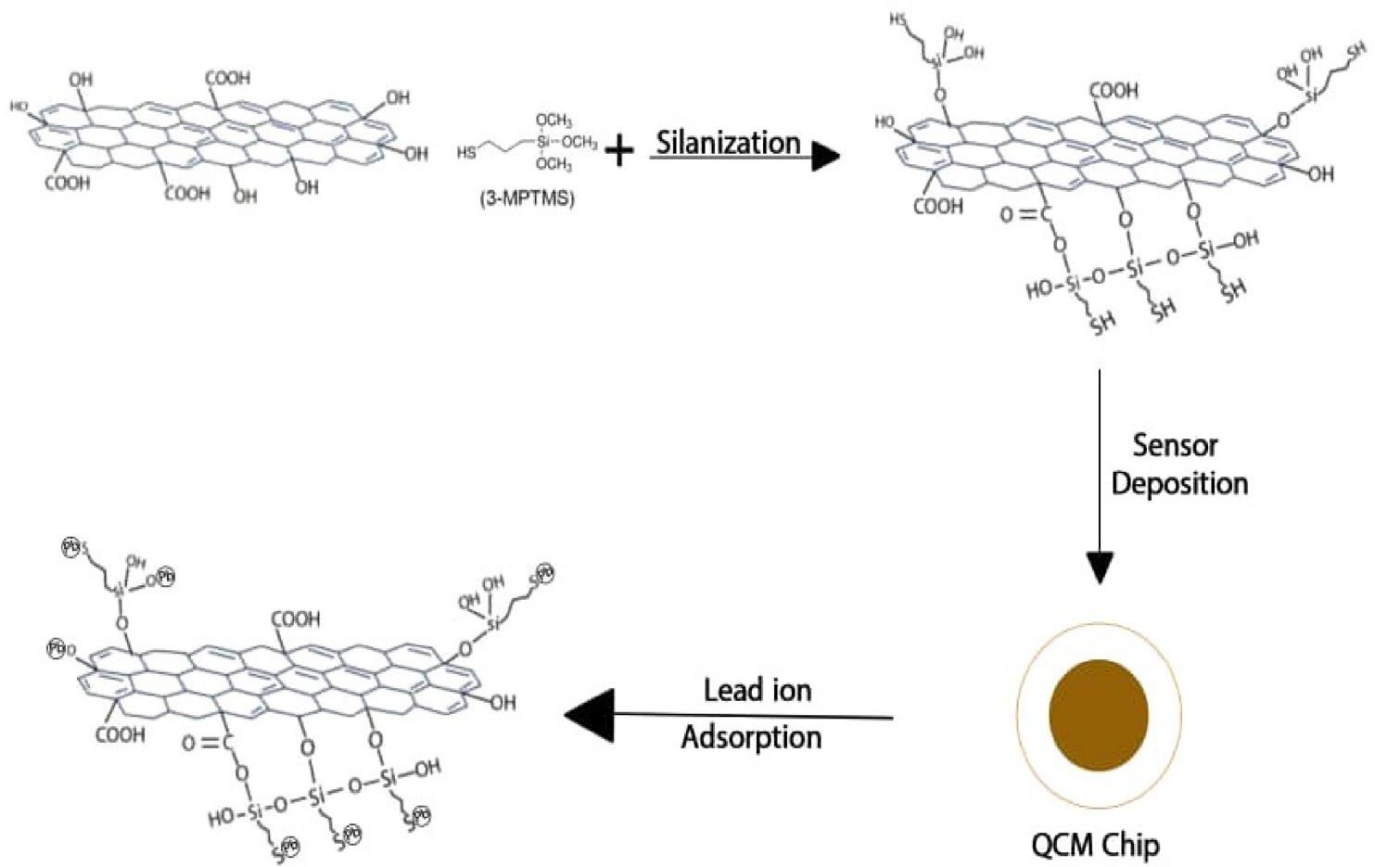
The pathway for functionalization of GO with 3-MPTMS and the possible mechanism of Pb^2+^adsorption on the 3-MPTMS-GO sensor.

The GO surface was functionalized by treating GO with the 3-MPTMS molecules (Scheme 1); 140 mg of viscous GO was added to a flask containing 350 mL of ethanol and ultrasonically diffused for 2 h. Thereafter, a freshly made 3% solution of 3-MPTMS was mixed with 15 ml of deionized water and added drop by drop to the flask while stirring for 12 to 14 h at 60 °C. Following the reaction’s conclusion, the flask was allowed to cool to ambient temperature before being diluted with more methanol. The dark brown powder known as 3-MPTMS-GO was produced by centrifuging the product, repeatedly washing it with a (60:40) acetone: water mixture and a (60:40) methanol: water mixture, then drying it at 70 °C for 12 h^35^.

### Instrumental

TEM using a Jeol-JEM-101 (Japan) was utilized to examine the structural properties of GO. Raman spectra were excited by 532 nm excitation wavelength using a WITec alpha 300 R confocal Raman microscope (Germany). The optical characteristics of the synthesized GO and 3-MPTMS-GO were assessed with a JASCO model V770 spectrophotometer. FT-IR spectra were obtained using a PerkinElmer Spectrum100 FT-IR device (US) within the range of 4000–500 cm^− 1^. The surface topography and roughness of the synthesized materials were visualized using AFM with a 5600Ls device manufactured by Agilent Technology (USA). XRD (EQUINOX 1000, Thermo Scientific Co., Lafayette, Co, USA) was used to affirm the formation of GO and 3-MPTMS-GO; the 2θ ranged from 5° to 80° with a scan speed of 0.1°/min. XPS: The results regarding surface charge properties and dispersion stability were measured with a zeta sizer (NanoSight NS500, Malvern Panalytical, Malvern, UK). The Biolin scientific contact angle analyzer (model T200) was used to assess the wettability of the surface.

### QCM technique

The QCM technique was utilized to evaluate the effectiveness of 3-MPTMS-GO as a sensor for detecting Pb^2+^ in water across various pH levels. A QCM (Q-senses, Biolin Scientific, Linthicum Heights, MD, USA) featuring gold electrodes was employed to perform this technique. The synthesized 3-MPTMS-GO sensor was suspended in ethanol at a concentration of 2 mg/ml and subsequently drop-cast onto the quartz crystal, using a volume of 50 µL, and allowed to dry at room temperature. The crystal was then positioned in the QCM flow cell. Prior to each experiment, the coated crystal was mounted in the QCM flow module, and the system was equilibrated with double-distilled water at a constant flow rate of 0.10 mL/min until a stable baseline was achieved (∆f < 1 Hz over 5 min). All measurements were conducted at a controlled temperature of 25 ± 0.1 °C. Following this, chemical solutions containing Pb^2+^ at concentrations of (0.05, 1, and 2) mg L^− 1^ were introduced to the surface of the 3-MPTMS-GO-based QCM sensor at different pH levels: 3, 7, and 12 in continuous flow mode. The lead solution was gradually introduced until a consistent signal was detected, signifying that equilibrium had been established in the binding interaction between the sensor and lead ions. A double-distilled water solution was added to the system after 2 min of exposure to eliminate non-adsorbed particles from the interfaces of the QCM sensor. For regeneration studies, the QCM chip was removed and immersed in 0.1 EDTA for 10 min, then rinsed with deionized water, and refixed in the flow cell for subsequent cycles.

## Results and discussion

### Surface and structural characterization of GO and 3-MPTMS-GO

#### TEM analysis

**Fig. 1:**
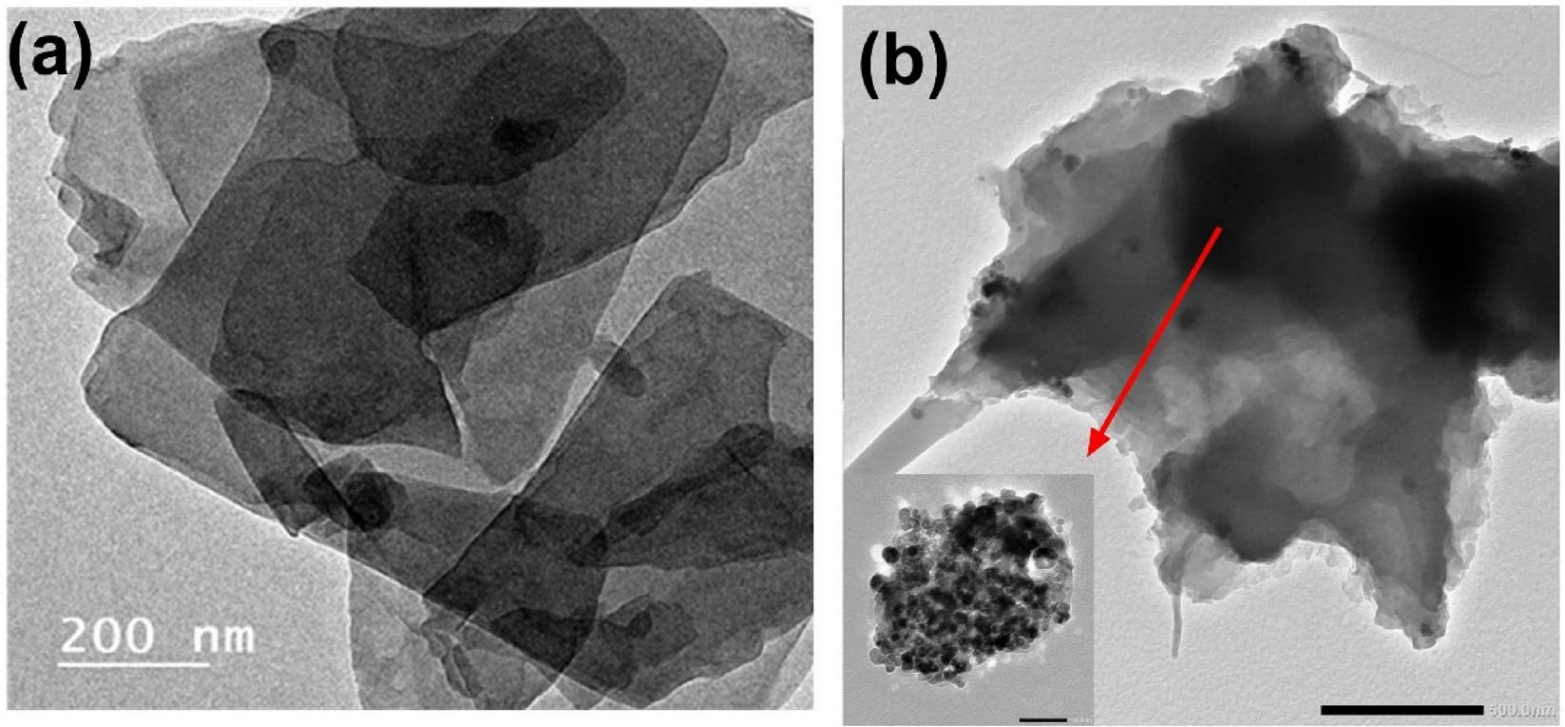
HRTEM image of (**a**) the prepared GO sheets and (**b**) 3-MPTMS-GO; the inset image shows functionalization regions with 3-MPTMS.

TEM analysis evaluates the characteristics of individual GO sheets, including their dimensions, form, and any observed rippling or folding on their surfaces^36^. The TEM analysis depicted in Fig. 1 reveals that the thinner regions indicate several layers of GO formed due to oxidation; the presence of oxygen-containing functional groups diminishes the van der Waals forces, promoting the exfoliation of the GO into thinner layers. Conversely, the darker sheets represent a tightly stacked nanostructure made up of some GO and/or graphene layers that have a limited number of oxygen-containing functional groups^37^. After functionalization of GO with 3-MPTMS as shown in Fig. 1b, it is observed that 3-MPTMS molecules have spherical morphologies and even homogenous spreading on the GO surface (inset), indicating successful functionalization of GO.

#### Raman spectroscopy analysis

**Fig. 2:**
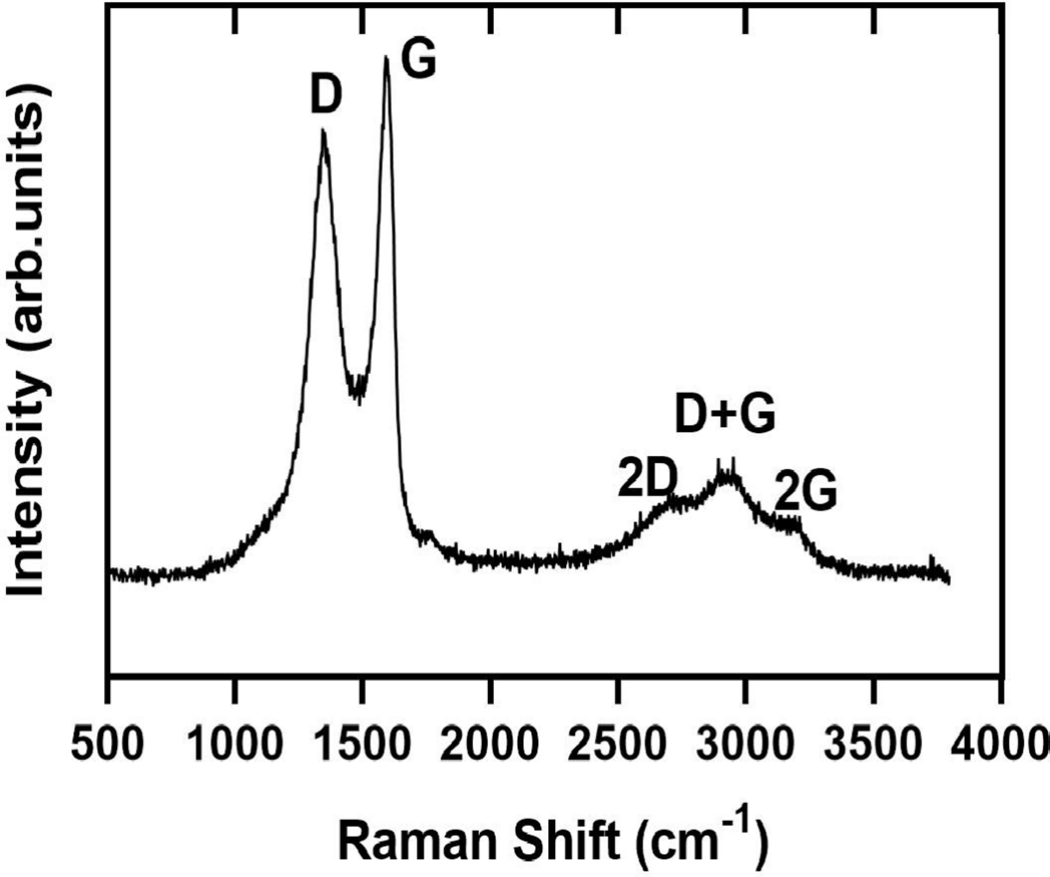
Raman spectra of GO sheets.

Raman spectroscopy is a non-invasive technique commonly utilized to extract structural insights about carbon-based materials^33^. The Raman spectra of GO sheets presented in Fig. 2 display two prominent peaks at 1355 cm^− 1^ and 1592 cm^− 1^, corresponding to the D and G bands, respectively. The D band results from the stretching vibration of sp^3^ carbon atoms, which is associated with oxidation. In contrast, the G band represents the stacking structure and originates from the stretching vibrations of sp^2^ carbon atoms, which are linked to the first-order scattering of the E_2g_ mode^38^. In the range of 2500 to 3200 cm^− 1^, second-order bands are observed, with the 2D band positioned at approximately 2716 cm^− 1^, which is the overtone of the D band due to a double-resonance mechanism^33,38,39^. The band at approximately 3197 cm^− 1^, referred to as the 2G band, is associated with the overtone of the G band, while the (D + G) band at around 2933 cm^− 1^ represents the combined overtone of both the D and G bands^39^. Quantitative analysis revealed an I_D/I_G ratio of 0.87 ± 0.04, calculated from integrated peak intensities after baseline correction. The Raman spectra indicate that the synthesis of GO has been successfully achieved.

#### UV–Vis spectroscopy study

**Fig. 3:**
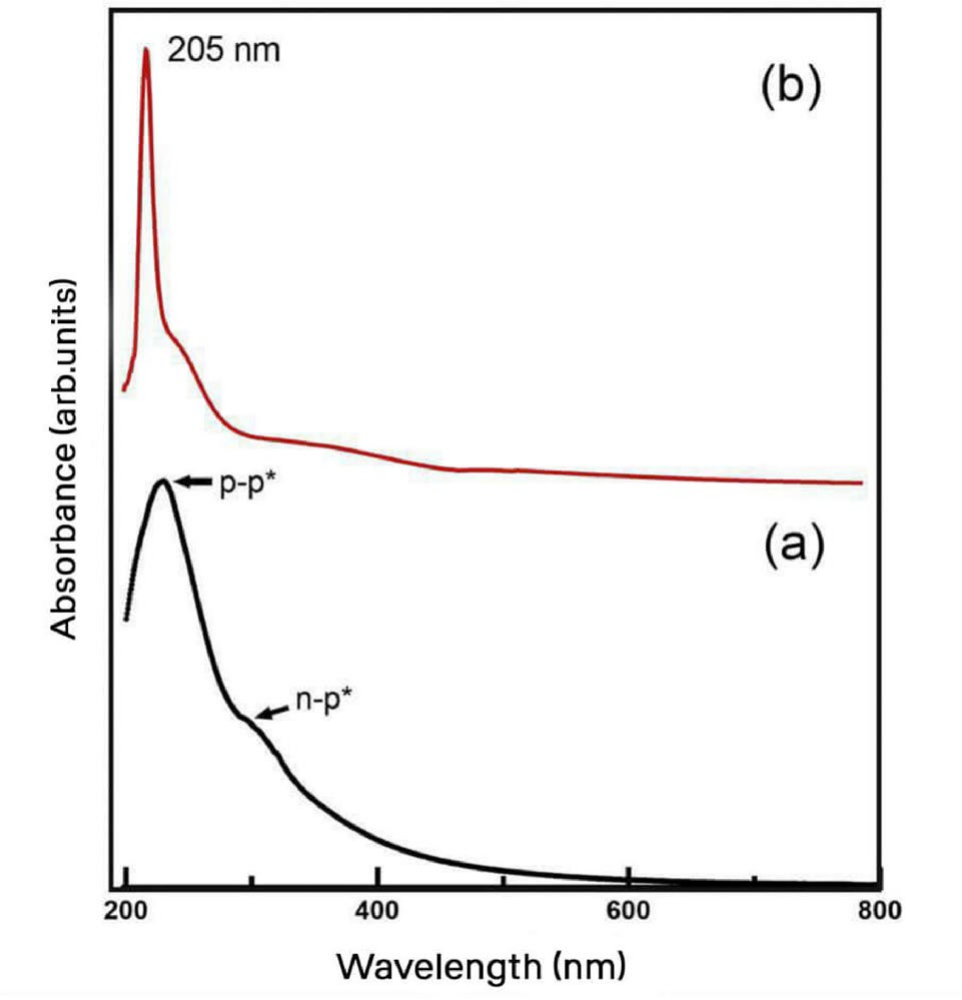
UV-Vis spectrum of aqueous dispersion of (**a**) GO and (**b**) 3-MPTMS-GO.

UV–Vis spectroscopy was utilized to assess the degree of oxidation and exfoliation^40^. Prior to analysis, distilled water was used for dispersion of GO and 3-MPTMS-GO. Figure 3a,b presents the UV–Vis spectrum of both GO and the 3-MPTMS-GO aqueous solution, respectively. In the spectrum of GO shown in Fig. 3a, two distinct peaks are observed. The shoulder around 301 nm can be attributed to the n − π* plasmon peak associated with C = O bonds, while the prominent peak at approximately 229 nm corresponds to the π − π* plasmon peak of aromatic C = C bonds^41^. Once GO has been functionalized with 3-MPTMS molecules, as shown in Fig. 3b, the primary aromatic peak C = C exhibits a blue shift, showing a strong peak around 205 nm^42^. Moreover, during the functionalization process, a decrease in the carbonyl peak is noted, indicating the attachment of 3-MPTMS molecules to GO^35,43^.

#### FT-IR study

**Fig. 4:**
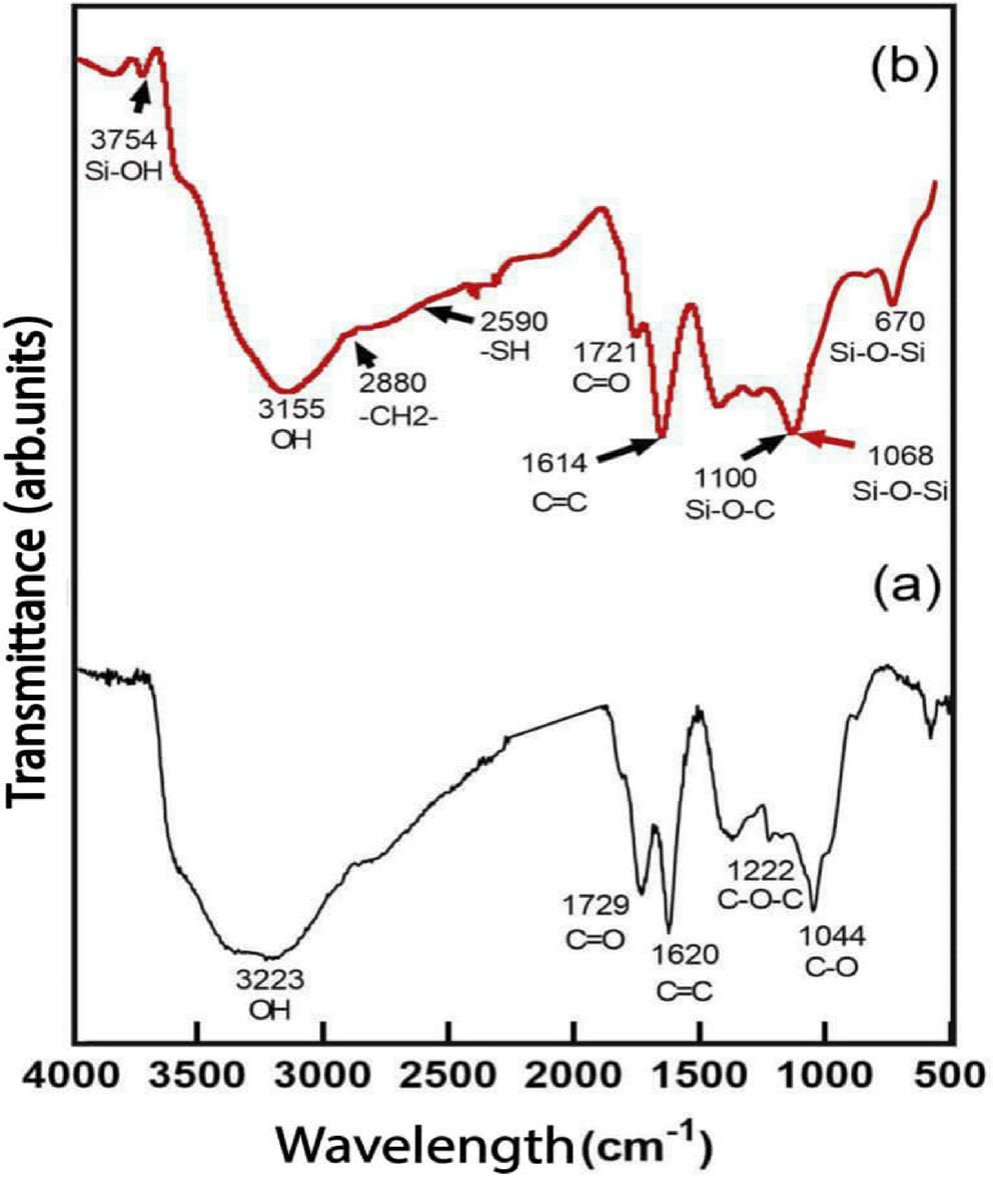
FT-IR spectra of (**a**) GO powder and (**b**) 3-MPTMS-GO powder.

To confirm successful functionalization, a study using FTIR was conducted on GO and 3-MPTMS–GO. As shown in Fig. 4a,b, the spectrum of GO powder depicted in Fig. 4a displays a prominent broad band near 3223 cm^− 1^, which can be attributed to the stretching vibration of hydroxyl groups (-OH). The oxygen-containing functional groups present in GO are identified through bands at approximately 1368 and 1729 cm^− 1^, corresponding to C-OH bending and C = O stretching vibrations of carboxyl groups (-COOH), respectively. The sharp band located at 1620 cm^− 1^ is linked to the C = C aromatic bond. The C-O stretching and C-O-C modes are differentiated at roughly 1044 and 1222 cm^− 1^, respectively. These peaks indicate the successful synthesis of GO^40,44^. Following the reaction with MPTMS, a pronounced broad band assigned to the stretching vibration of hydroxyl groups (-OH) shifts to right near 3155 cm^− 1^, new spectral peaks appeare in the 3-MPTMS-GO spectra, as illustrated in Fig. 4b. The prominent peak at 1721 cm^− 1^, linked to the C = O stretching of -COOH groups, showed a reduction in intensity, which corroborates the findings from the UV-Vis results. The previously observed peak at approximately 1044 cm^− 1^ was replaced by several new significant peaks, with Si-O-C appearing at around 1100 cm^− 1^ and Si-O-Si asymmetric stretching noted at roughly 1068 cm^− 1^, both of which are associated with the Si-O-Si backbone structure^45^. The faint peak at about 2590 cm^− 1^ corresponds to the S-H stretching vibration^30^. A newly identified prominent peak at approximately 2880 cm^− 1^ can also be associated with the asymmetrical -CH_2_- group present in 3-MPTMS^43^, further confirming the successful attachment of SH groups onto the GO. The distinct peak around 670 cm^− 1^ reflects Si-O-Si bending and symmetrical stretching^46,47^. The latest peak observed at 3754 cm^− 1^ pertains to silanol groups (-Si-OH)^48^. The appearance of these additional peaks signifies the successful functionalization of GO. The process of functionalizing GO through a cross-linking reaction with 3-MPTMS occurs in two stages. Initially, in the first stage, 3-MPTMS is hydrolyzed in water, transforming the methoxy groups into -Si-OH groups. In the second stage, the -Si-OH groups engage with the -OH and -COOH groups present on the surface of GO via a condensation reaction, leading to the creation of silane chains. These mechanisms correspond closely with prior experiments^30,49^.

#### XRD analysis

**Fig. 5:**
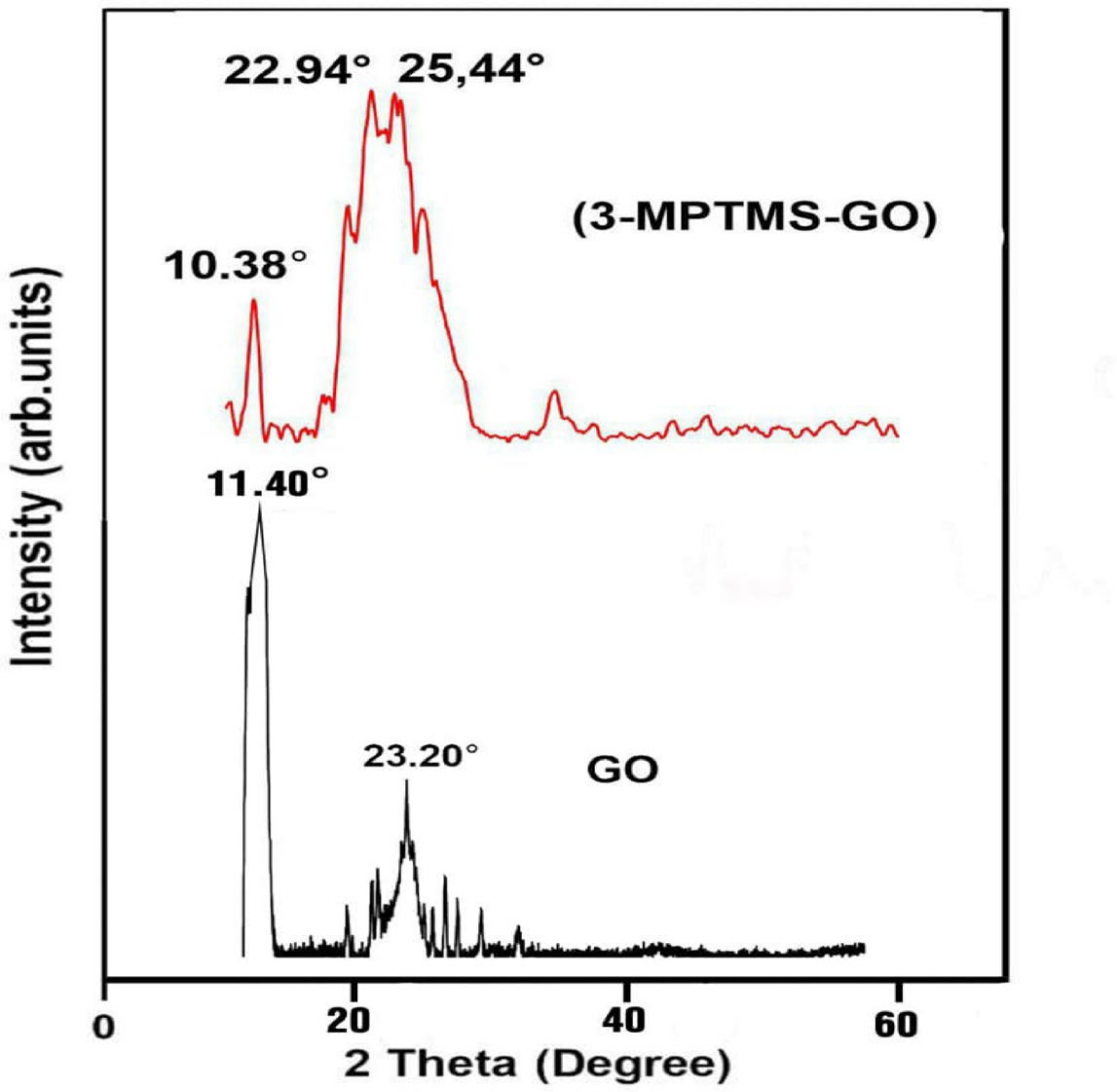
XRD curves of GO and 3-MPTMS-GO.

The XRD analysis is used to validate the above structural merits. As displayed in Fig. 5, the XRD spectrum of GO shows a strong diffraction peak at 11.40° corresponding to the (001) plane and d-spacing of 0.776 nm, likely due to the uneven assembly of the GO sheets^50^. Following functionalization of GO with 3-MPTMS, the XRD spectrum of 3-MPTMS-GO displays sharp diffraction peaks at 10.38°, relating to the (001) plane and d-spacing of 0.851 nm, and broad peaks at 22.94° and 25.44°, corresponding to (002) planes, respectively. These results suggest a distinguished increase in interlayer spacing, representing a 9.7% interlayer expansion which can be assigned to the presence of the grafted 3-MPTMS molecules between the GO sheets^51^. which corroborates the FTIR findings that different silane molecules can be effectively attached to the GO sheets.

#### AFM analysis

**Fig. 6:**
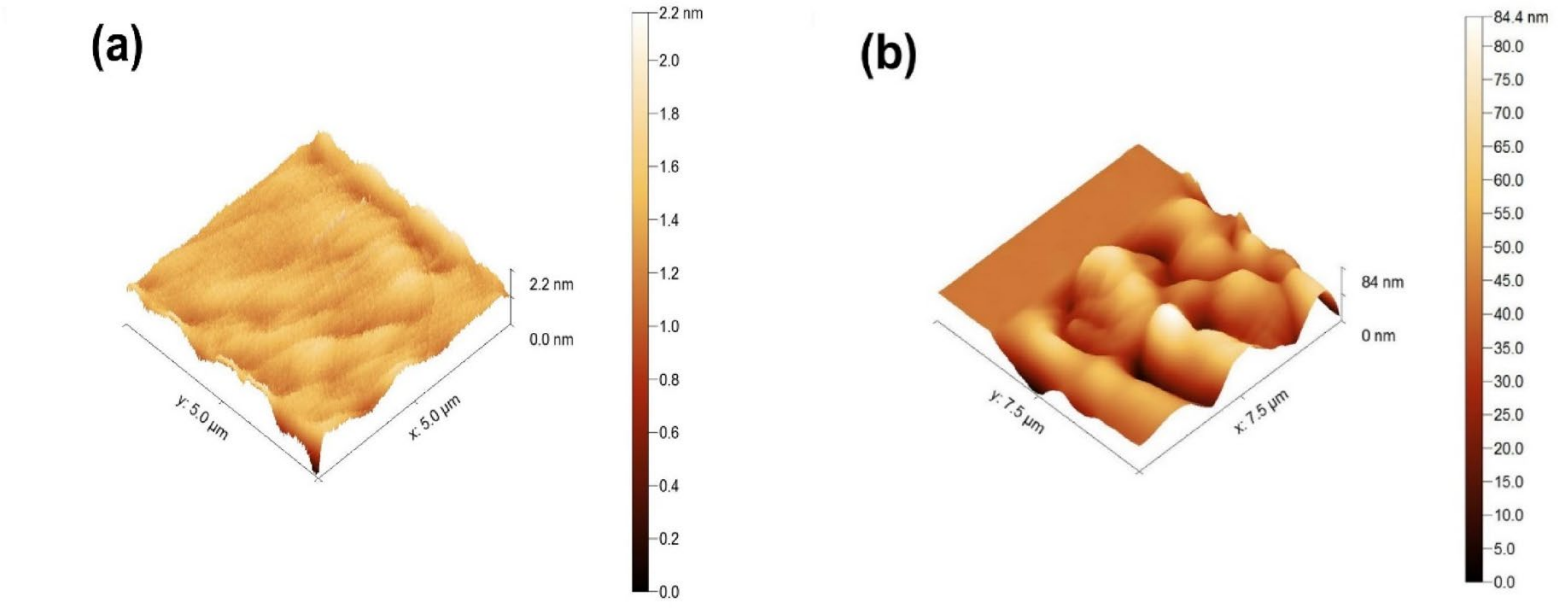
3D AFM image of (**a**) synthesized GO and (**b**) 3-MPTMS-GO.

The AFM was employed to evaluate the surface roughness and thickness of GO and 3-MPTMS-GO^49^. The AFM technique is based on the interactions between the probe of the instrument and the surfaces of the GO and 3-MPTMS-GO^52^. Figure 6a,b displays three-dimensional (3D) AFM images of GO and 3-MPTMS-GO, respectively. As shown in Fig. 6a, the analysis indicated that the GO sheets exhibited a smooth surface morphology with a height of about 2.2 ± 0.07 nm, which corresponds to few-layer GO^53^. The root mean square surface roughness (RMS) of GO was determined to be 0.15 ± 0.01 nm. Figure 6b illustrates the surface topography of 3-MPTMS-GO. It is evident that 3-MPTMS-GO exhibits irregularities and a surface structure with an average height of about 84.4 ± 1.6 nm. As a result, the RMS of 3-MPTMS-GO was estimated to be 11.24 ± 0.37 nm. The 75-fold increase in surface roughness corresponds to a surface area ratio increase from 1.02 to 1.89. From this, we can infer that the thickness of the synthesized 3-MPTMS-GO sheet exceeds that of the exfoliated GO, which can be attributed to the presence of functionalized silane chains that are bonded to the GO^54,55^. The considerable increase in surface roughness of 3-MPTMS-GO results in a larger surface area and enhanced adsorption capacity, which is especially advantageous for the detection of heavy metals.

#### Contact angle, hydrophilicity evaluations

**Fig. 7:**
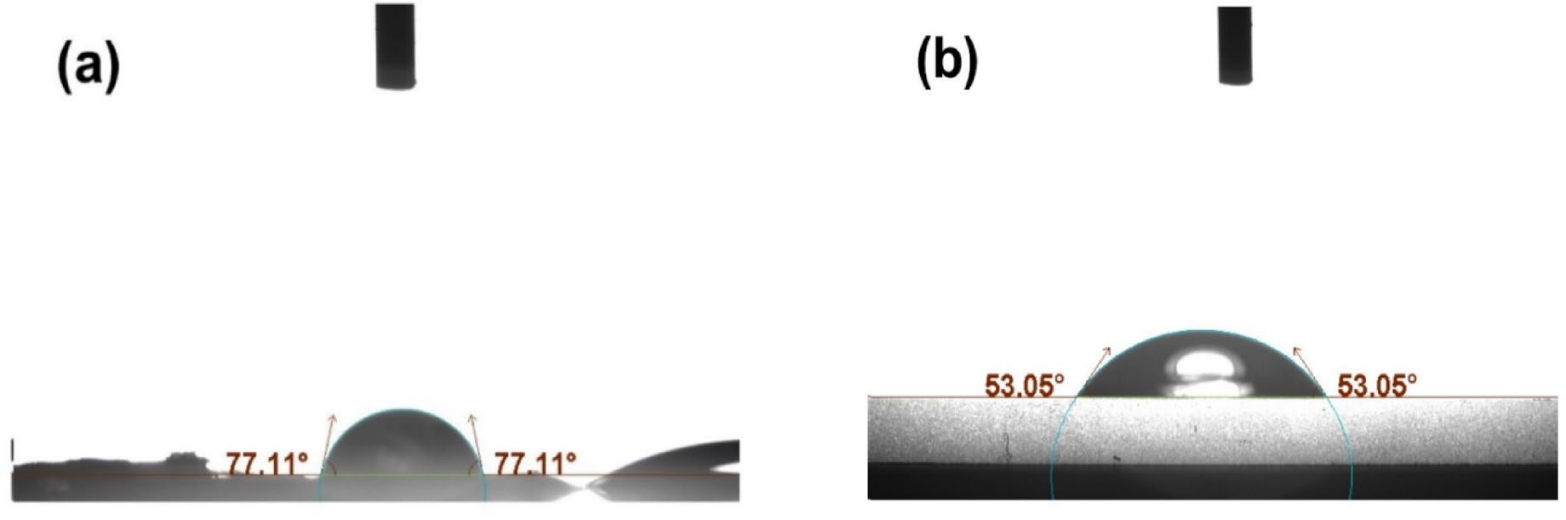
The contact angle (**a**) between GO and water and (**b**) between 3-MPTMS-GO and water.

The level of hydrophilicity of a material can be determined by measuring the contact angle between its solid and liquid phases^56^. As shown in Fig. 7a,b, the contact angle measurements reveal that GO has a contact angle of 77.11°, whereas 3-MPTMS-GO exhibits a contact angle of 53.15°. The decrease in the contact angle of GO from 77.11° to 53.15° indicates that the introduction of 3-MPTMS molecules enhances the hydrophilicity of its surface. According to Wenzel’s law, increasing the roughness of a hydrophilic surface leads to a reduction in the contact angle^57,58^. Therefore, the reduced contact angle of 3-MPTMS-GO is thought to be a result of its greater surface roughness. This observation is consistent with the findings from the AFM analysis.

#### Zeta potential measurements

Zeta potential is a significant physicochemical property that reflects the stability of nanoparticles. Extreme values of Zeta potential, whether high or low, result in strong repulsive forces, and repulsion between similarly charged particles prevents aggregation, making dispersion easier^59^. The Zeta potential of the synthesized 3-MPTMS-GO at pH 7.0 and 25 °C is −31.12 mV. The FT-IR analysis suggests that negatively charged groups like -OH, -COOH, -SH, and -Si-OH play a crucial role in providing the surface with a negative charge^60^. This negative Zeta potential indicates that 3-MPTMS-GO exhibits excellent dispersion stability and is suitable for detecting positively charged ions in an aqueous medium.

### Detection of Pb²⁺ by 3-MPTMS-GO-based QCM sensor

The methods for detecting heavy metals using a QCM apparatus provide real-time insights into the mechanical response of a 3-MPTMS-GO sensor to Pb²⁺ present on the QCM chip’s surface. The QCM chip features a quartz crystal with gold electrodes coated on it; when nanogram amounts of an external substance are introduced onto the quartz crystal surface, the resonance frequency (f) experiences a shift. This change in resonance frequency (Δf) is closely linked to the mass variation, facilitating highly accurate mass measurements through the Sauerbrey equations^61,62^.

1 $$\Delta m\,=\, - \,C * 1/n * \Delta f$$

Where (∆m) is the mass change (ng), (C) is a constant that depends on the crystal’s thickness and other characteristics, (n) is the overtone number (the crystal’s working area), and (∆f) is the measured frequency change.

The hydrophilic properties and structural stability of the synthesized 3-MPTMS-GO enhance the adhesion of thin films to the surface of the gold electrode. The strong interaction between the sensor materials that were synthesized and the gold electrode on the quartz crystal guarantees the stability of the thin film in water, which ultimately improves the coating of the QCM detector. As a result, the deposition of 3-MPTMS-GO on the surface of the QCM chip allows for the real-time detection of Pb²⁺ taken from an aqueous solution while managing the pH of the solution through a QCM technique.

#### Effect of pH

**Fig. 8:**
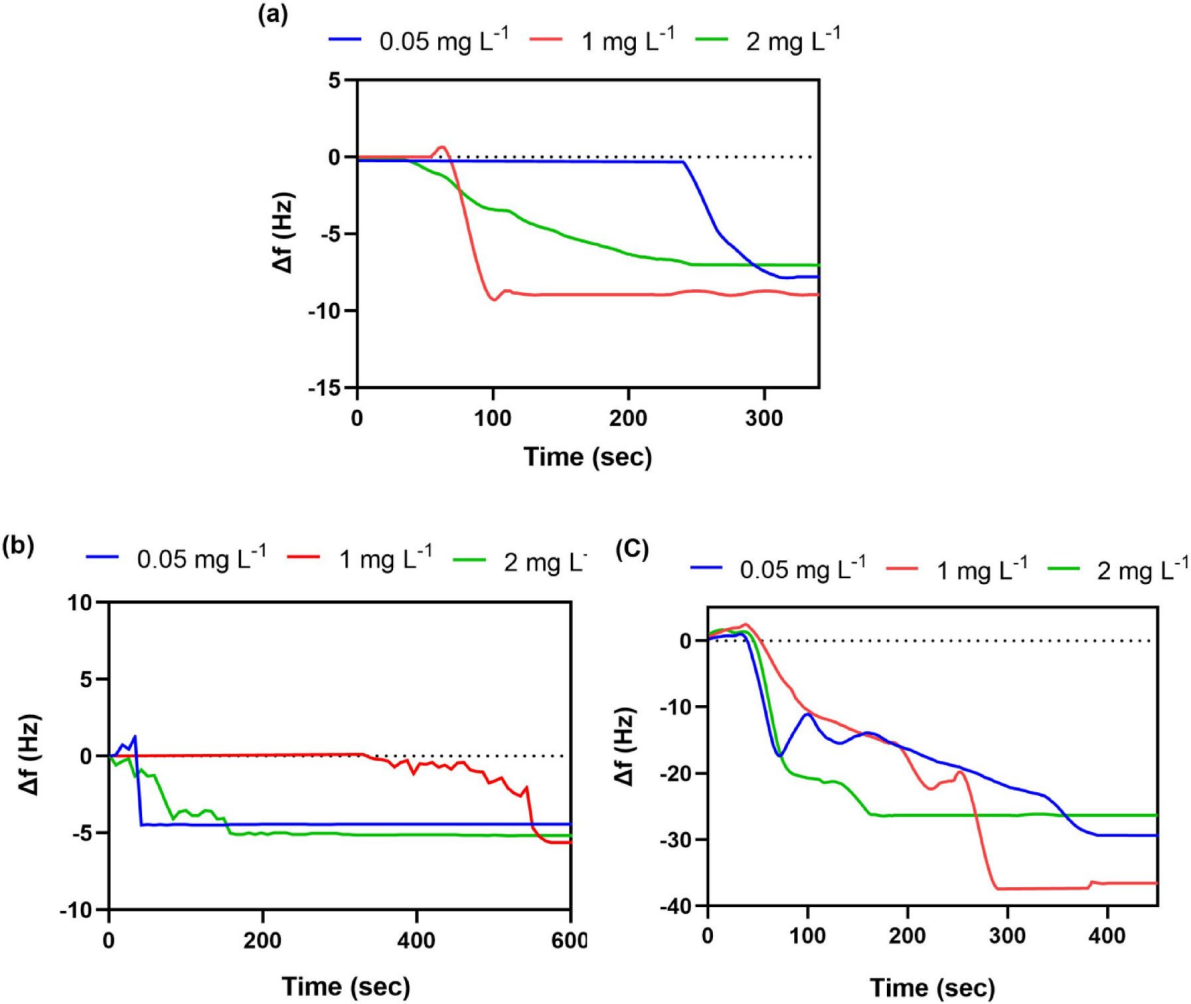
Real-time QCM frequency change (Δf) at Pb²⁺ concentrations 0.05, 1, and 2 mg L^− 1^ and pH values: (**a**) pH 7, (**b**) pH 3, (**c**) pH 12, recorded by the 3-MPTMS-GO sensor.

Figure 8 illustrates the changes in frequency (∆f) measured by the 3-MPTMS-GO sensor in response to Pb²⁺ concentrations of 0.05 mg L^−1^, 1 mg L^−1^, and 2 mg L^−1^ across different pH levels of 3, 7, and 12. Under neutral conditions, where the pH is neutral, the 3-MPTMS-GO sensor established a notable decline in ∆f when exposed to Pb²⁺. At a concentration of 0.05 mg L^−1^ of Pb²⁺, the graph designates a drop in frequency, which reaches ∆f = − 7.70 ± 0.50 Hz after approximately 3 min before stabilizing. This frequency drop can be attributed to the binding of Pb²⁺ to the active sites on the sensor’s surface. As shown in Fig. 8a, the ∆f values measured were − 8.95 ± 0.90 Hz at 1 mg L^−1^ and − 7.08 ± 0.09 Hz at 2 mg L^−1^. It is evident that the frequency response measured by the 3-MPTMS-GO sensor decreases gradually with increasing Pb²⁺ concentration up to 1 mg L^−1^. This observation can be attributed to the availability of active sites at lower concentrations, where the majority of Pb²⁺ are effectively absorbed and identified. Conversely, as the concentration increased to 2 mg L^−1^, more Pb²⁺ filled the available active sites, resulting in saturation of these sites; consequently, the detection of Pb²⁺ became restricted, and the frequency response diminished^63^. At a pH level of 3, the 3-MPTMS-GO sensor revealed quite good sensitivity to Pb²⁺ ions as shown in Fig. 8b, where ∆f values of − 4.43 ± 0.40 Hz, − 5.75 ± 0.70 Hz and − 5.20 ± 0.25 Hz were obtained at Pb²⁺ concentrations of 0.05 mg L^−1^,1 mg L^−1^, and 2 mg L^−1^, respectively. This reduced sensitivity discerned in comparison to neutral conditions could be accounted to the slightly elevated levels of H^+^ ions in the solution that compete with Pb²⁺ for the available active adsorption sites (protonation of functional groups), leading to a reduction in the binding sites reachable for Pb²⁺^40^. At a pH level of 12 shown in Fig. 8c, where ∆f values of − 29.53 ± 0.60 Hz, − 36.55 ± 0.50 Hz and − 26.53 ± 0.55 Hz were attained at Pb²⁺ concentrations of 0.05 mg L^−1^,1 mg L^−1^, and 2 mg L^−1^, respectively. At alkaline conditions (pH > 11), the Pb^2+^ species are present in the form of Pb (OH)_2_ formed as a precipitate in solution and Pb (OH)^-^_3_. Extra -OH groups in solution combine with -Si-O-Si- present on the sensor forming the Si-OH compound (deprotonation of functional groups). This compound condenses with Pb (OH)^-^_3_ in solution to form Si-O-Pb through a chemisorption process. Thus, the Si-OH group acts as an adsorption site along with other adsorption sites such as -SH, -OH, and -COOH groups^64,65^. These findings demonstrate a slight effect of pH on sensor response at acidic medium due to protonation of binding sites. By increasing pH value (> 7) at alkaline conditions, the 3-MPTMS-GO sensor exhibits exceptional sensitivity introduces due to abundant available adsorption sites for Pb²⁺ that leads to, particularly in alkaline conditions.

#### Calibration curve and LOD calculation

**Fig. 9:**
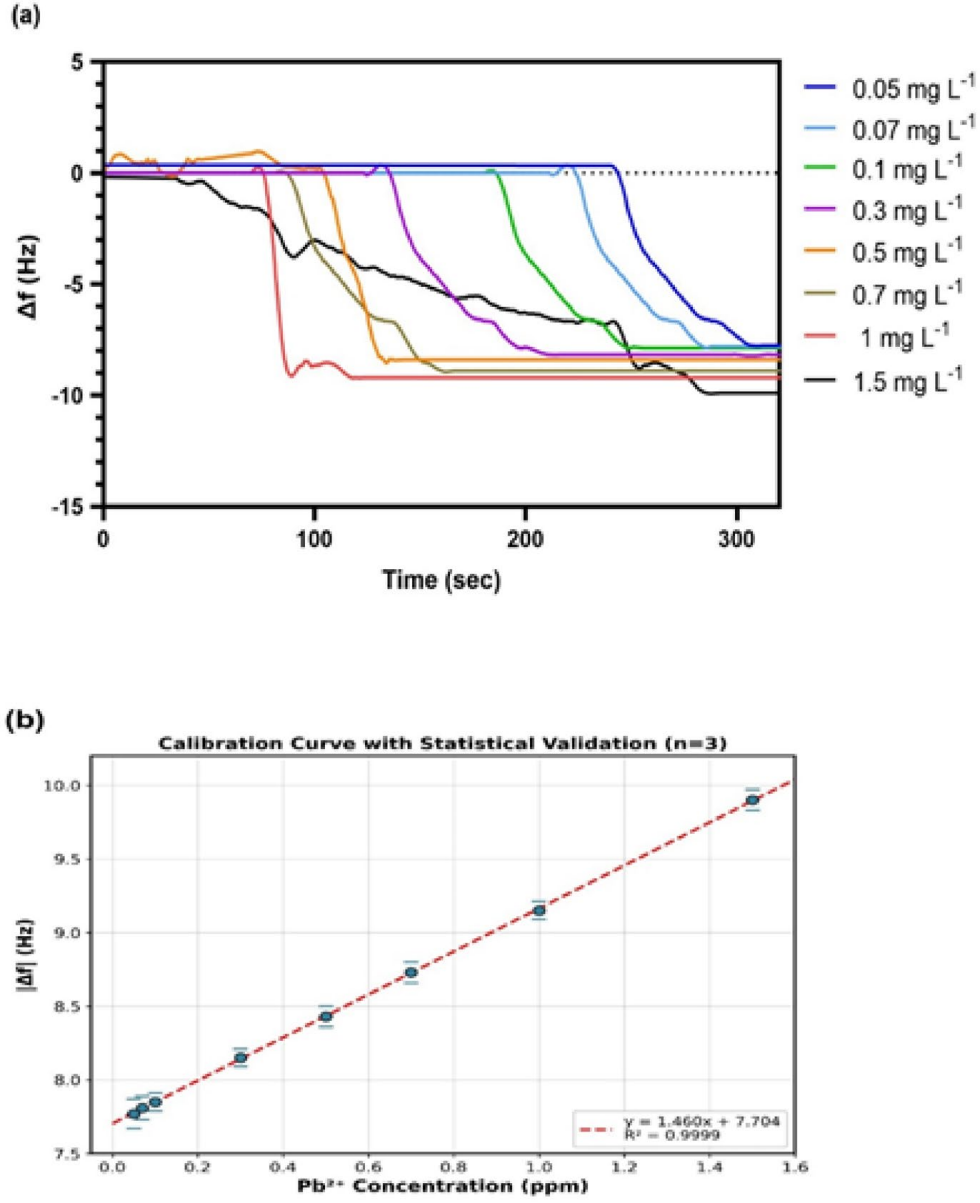
Detection of Pb²⁺ across (0.05–1.5 mg L^−1^) concentration range by the 3-MPTMS-GO-based QCM sensor (**a**) Concentration-dependent response, (**b**) Calibration curve with statistical validation.

The real-time responses of the 3-MPTMS-GO sensor towards Pb^2+^ across a (0.05–1.5 mg L^− 1^) concentration range were measured at a pH adjustment of (7.0 ± 0.1), calibration at neutral pH provides practically relevant analytical parameters, matching WHO drinking water guidelines It was reported that the response increased gradually from lower to higher concentrations of Pb^2+^ as displayed in Fig. 9a. A comprehensive calibration study has been conducted using a broad concentration range of Pb^2+^ (0.05–1.5 mg L^− 1^) in order to find the possible detection limit (Table 1S). The analysis reveals that the 3-MPTMS-GO sensor establishes a linear range from 0.05 mg L^− 1^ to 1.5 mg L^− 1^ where the linear regression equation towards Pb^2+^ from the calibration curve was y = 1.460x + 7.704 (R² = 0.9999) as shown in Fig. 9b and for logarithmic response was y = 1.204x + 9.133 (R² = 0.8358) as depicted in (Fig. 2S). Where y is the frequency response (|Δf|) of different concentrations, x is concentration of Pb^2+^ and R² is regression co-efficient. The LOD was calculated as 3σ/S = 3(0.005)/1.460 = 0.01 mg L^− 1^, where σ is the standard deviation of the blank response (*n* = 10, blank SD = 0.005 Hz) and S is the sensitivity (slope = 1.460 Hz/mg L^− 1^). This LOD corresponds to the WHO regulatory limit for Pb²⁺ in drinking water. Statistical validation was performed with triplicate measurements at each concentration point (*n* = 3). The relative standard deviation (RSD) ranged from 0.66% to 1.29%, demonstrating excellent measurement precision. Inter-sensor reproducibility was evaluated using five independently prepared sensors. The RSD values (< 1% for response measurements) confirm excellent batch-to-batch consistency (Table 2S) (Fig. 2S). As Table 1 illustrates, the 3-MPTMS-GO-based QCM sensor verified a remarkably low LOD value compared to other formerly informed methods such as electrochemical sensors (LOD value: 0.033), which need complicated steps of synthesis, including the use of costly materials, and are therefore time-consuming^66^. Our selection of 3-MPTMS offers several practical advantages for QCM sensor development. In terms of synthesis and functionalization, 3-MPTMS enables single-step functionalization through the direct silanization of GO. For stability and performance, 3-MPTMS provides stable thiol-metal bonds through wide-ranging pH levels suitable for environmental samples. So, this study demonstrates the effectiveness of the 3-MPTMS-GO-based QCM sensor as a sensitive platform that uses low-cost materials, simple instrumentation, and portable detection equipment for monitoring Pb²⁺ compared to other methods.

The comparison with the electrochemical detection method highlights the complementary advantages of the QCM methodology. While electrochemical sensors depend on electron transfer kinetics and redox activity at electrode surfaces, the QCM operates through a mass sensitive method that enables real-time quantification of Pb²⁺ and tracks changes in mass immediately without requiring electroactivity. This advantage allows QCM sensors to function efficiently even in samples exhibiting inadequate electrochemical performance and reveals information on kinetics, mechanism that are invisible to electrochemical sensors. Moreover, QCM overcomes conventional drawbacks of electrochemical sensors, such as electrode contamination, instability, and signal drift. Consequently, the amalgamation of 3-MPTMS-GO with QCM enhances both sensitivity and selectivity for Pb²⁺, while also providing a dependable alternative to more complex redox-based electrochemical detection methods.

**Table 1:** Comparison of a 3-MPTMS-GO-based QCM sensor with other methods in the literature used for lead ion detection.

Detection method	LOD (mg L^− 1^)	Detection range (mg L^− 1^)	Preparation method	Real samples	Reference
CoFe2O4/Ca-alginate nanocomposite-based QCM sensor	(0.000125)	Not reported	Green synthesis of ferrite nanoparticles + biopolymer encapsulation	Not reported	^17^
Electrochemical sensors using MOF-5/PANI electrodes	(0.033)	0.63–3.7	MOF fabrication + PANI polymerization	water samples	^66^
Fluorescent sensor based on functionalized MOF bioprobe	(0.0095)	0.05–1 Narrow range	Functionalized of MOF with bioprobe molecules	Tap water and River water	^67^
Plasmon resonance sensor based on organometallic Pt^2+^	(0.05)	Not reported	Synthesis of Pt-complex+ immobilization on SPR chip	Not reported	^68^
Electrochemical sensors using GCE/MXene/Nafion modified electrode	(0.0097)	0.03–0.20	MXene synthesis + electrode modification with Nafion	Not reported	^69^
3- MPTMS functionalized GO -based QCM sensor	(0.01)	0.05–1.5	Simple salinization + drop casting	Industrial, ground water, tap water	This study

#### Adsorption isotherm and kinetic analysis

To elucidate the adsorption mechanism, the sensor response data were analyzed using Langmuir isotherm and pseudo-second order kinetic models. The linearized Langmuir equation (C/Δf = 1/(K_L_ × Δf _max_) + C/Δf _max_) was applied to the equilibrium data. The excellent fit (R² = 0.9987) yielded Δf _max_ = 2.52 Hz and K _L_ = 0.1825 mg L^− 1^, indicating monolayer adsorption on a homogeneous surface consistent with specific Pb²⁺-thiol coordination at discrete active sites (Fig. 3S). The linearized pseudo-second order equation (t/q_t_ = 1/(k₂ × q^2^_e_) + t/q_e_) was applied to time-dependent data. Parameters obtained: q_e_(calc) = 9.25 Hz (experimental: 9.15 Hz), k₂ = 0.0892 min⁻¹·Hz⁻¹, R² = 0.9995. The excellent fit confirms chemisorption as the rate-limiting step, consistent with strong Pb²⁺-thiol coordination bonds (Fig. 4S). These isotherm and kinetic analyses support the proposed sensing mechanism involving specific coordination of Pb²⁺ at thiol functional groups, rather than non-specific physisorption.

### Interferences effect and selectivity study

**Fig. 10:**
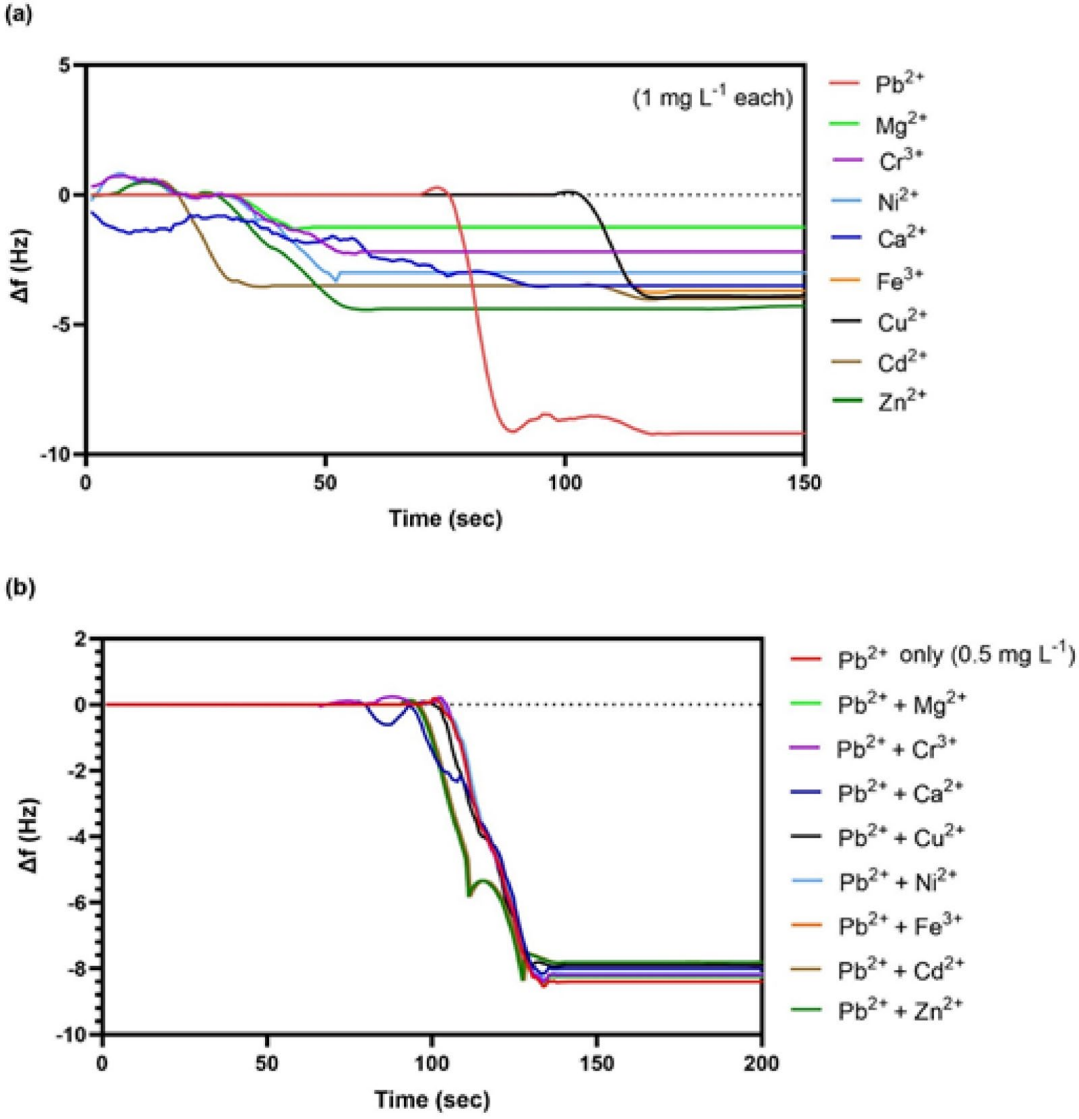
(**a**) Real-time QCM frequency responses for individual metal ions: Pb^2+^, Zn^2+^, Cu^2+^, Ca^2+^, Ni^2+^, Fe^3+^, Mg^2+^, Cd^2+^ and Cr^3+^ (1 mg L^− 1^ each) at neutral pH (**b**) Sensor response to Pb^2+^ (0.5 mg L^− 1^) in the presence of interfering ions (2 mg L^− 1^ each), showing 93–98% signal retention compared to the detection of Pb^2+^ only (0.5 mg L^− 1^).

Here, interference effect evaluation is one of the significant sensor processes in order to determine the effect of environmentally relevant metal ions, such as Zn^2+^, Cu^2+^, Ca^2+^, Cd^2+^, Ni^2+^, Fe^3+^, Mg^2+^, and Cr^3+^, on the results of Pb^2+^ detection. The real-time response of the 3-MPTMS-GO sensor was assessed by measuring individual ion response at equal concentrations of 1 mg L^− 1^ and pH (7.0 ± 0.1). As depicted in Fig. 10a, the frequency change (∆f) reaches to − 9.28 ± 0.2 Hz for Pb^2+,^ compared to the frequency changes for the interfering metal ions: Zn^2+^ (− 4.30 ± 0.4 Hz), Cu²⁺ (− 3.95 ± 0.5 Hz), Ca²⁺ (− 3.50 ± 0.5 Hz), Ni^2+^ (− 3.05 ± 0.1 Hz), Fe^3+^ (− 3.73 ± 0.6 Hz), Cd^2+^ (− 4.15 ± 0.2 Hz), Mg^2+^ (− 1.25 ± 0.3 Hz), and Cr³⁺ (− 2.23 ± 0.3 Hz). To evaluate the anti-interference ability of the 3-MPTMS-GO sensor, mixed-ion studies were made using binary mixtures of Pb²⁺ (0.5 mg L^-^^1^) with 4-fold excess metal ions: Zn^2+^, Cu^2+^, Ca^2+^, Cd^2+^, Ni^2+^, Fe^3+^, Mg^2+^ and Cr^3+^ as shown in Fig. 10b. The findings revealed 93–98% signal retention (Table 3S) (Fig. 5S). The interference effect was evaluated across three Pb²⁺ concentration levels (0.01, 1.0, and 2.0 mg L^− 1^) representing the WHO limit, working range, and upper detection range. Signal recovery remained > 90% across all levels (Table 4S), consistent with reported thiol-based sensors in literature^29^, confirming robust selectivity independent of analyte concentration. All the results indicated that the 3-MPTMS-GO sensor possessed outstanding selectivity for Pb^2+^ in the existence of various foreign metal ions with high concentrations. The exceptional selectivity of the 3-MPTMS-GO sensor stems from preferential thiol-Pb²⁺ coordination chemistry, where Pb^2+^ exhibits strong affinity for the thiol group grafted on the sensor’s surface according to HSAB theory, supported by the measured Zeta potential (−31.12 mV) providing additional electrostatic selectivity. It is clear that the synthesized 3-MPTMS-GO-based QCM sensor is suitable for selective detection of Pb^2+^ while maintaining considerable sensitivity; however, widespread interference analyses are required before practical implementation for environmental monitoring applications.

### Real sample analysis

The analysis encompasses a group of real samples (industrial effluent from metal plating and electronics facilities (*n* = 3), groundwater samples from various locations (*n* = 4), and municipal tap water samples from different Cairo districts (*n* = 5) to validate the synthesized 3-MPTMS-GO-based QCM sensor. Prior to detection, the real samples were filtered using 0.45 μm membrane filtration to remove suspended solids and pH was adjusted through (5.6–7.3) range. Each sample was introduced into the QCM flow cell and analyzed using a 3-MPTMS-GO sensor (2 mg/ml and 50 µL). As shown in (Fig. 6S), the 3-MPTMS-GO sensor exhibits real-time responses for each sample type, demonstrating rapid equilibration and a stable signal plateau. Then, a standard solution of Pb^2+^ was spiked into real sample solutions for recovery evaluation using the spike-recovery method. The results indicated that the recovery range of Pb^2+^ was 103 − 92%. (Table 5S). Baseline Pb²⁺ concentrations were independently verified by ICP-MS analysis prior to QCM measurements. All the findings show that the measurements of Pb^2+^ in real samples by the 3-MPTMS-GO-based QCM sensor were slightly affected with variation real sample and confirm that the sensor is reliable and appropriate for analyzing various real environmental samples.

### Sensor regeneration and stability evaluation

Sensor regeneration was achieved simply by immersing the QCM crystal in a 0.1 M EDTA solution for 10 min and rinsing with deionized water. EDTA (ethylenediaminetetraacetic acid) is a powerful aminopolycarboxylate chelating ligand (log K = 18) that rapidly removes Pb²⁺ from the surface during regeneration, restoring active sites without harming the functional layer^70^. (Fig. 7Sa) shows the detection efficiency of Pb²⁺ by the 3-MPTMS-GO sensor with 10 cycles. The detection efficiency remained above 94%, even in the tenth cycle. This ability to regenerate allowed for the recovery of the original sensor sensitivity within 10 consecutive cycles, highlighting the sensor’s recyclability and durability. Stability of the synthesized 3-MPTMS-GO was scrutinized according to its reply to 1 mg L^− 1^ of Pb²⁺ at neutral conditions for up to 14 days. After 2 days as shown in (Fig. 7Sb), a slight decline in the efficiency response of the 3-MPTMS-GO sensor as 99.6% with a corresponding ∆f value of − 9.24 ± 0.50 Hz is observed, suggesting nominal degradation. The 3-MPTMS-GO sensor maintained about 96% of its initial efficiency after 14 days, signifying respectable stability over time. The findings on stability and regeneration support the high economic value of the 3-MPTMS-GO sensor.

### Quantitative XPS survey spectra analysis and adsorption mechanism interpretation

#### XPS survey spectra analysis

**Fig. 11:**
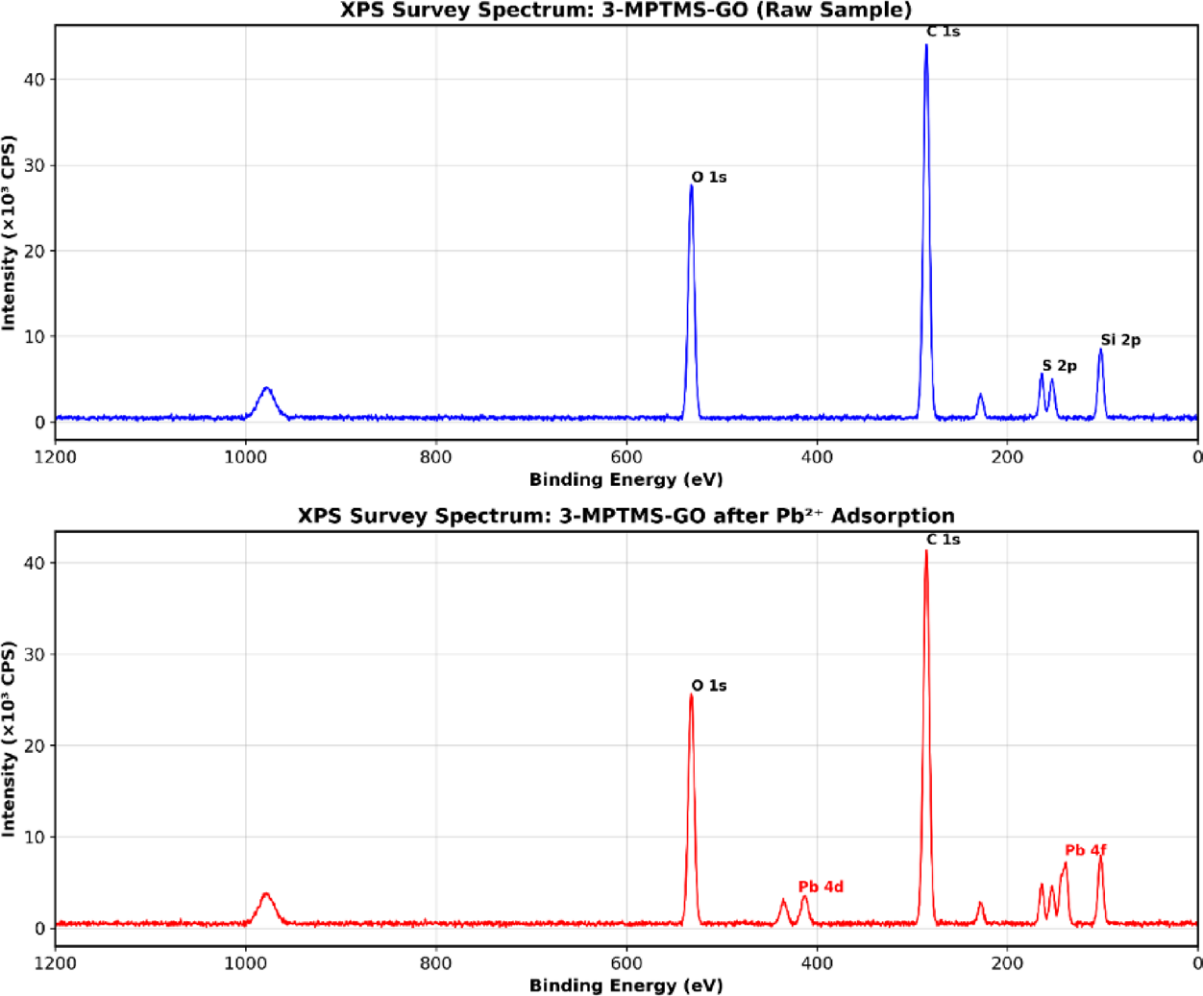
XPS survey spectra comparison of 3-MPTMS-GO before (blue line) and after (red line) Pb²⁺ adsorption.

XPS was employed to verify the surface chemical composition and confirm successful Pb²⁺ adsorption on the 3-MPTMS-GO sensor. XPS survey spectra were recorded for both the raw 3- MPTMS-GO sample and the 3-MPTMS-GO sample following exposure to Pb²⁺ solutions, as presented in Fig. 11.

As depicted in Fig. 11, the XPS survey spectrum of raw 3-MPTMS-GO displays characteristic peaks corresponding to carbon (C 1 s) at approximately 285 eV, oxygen (O 1 s) at ~ 533 eV, silicon (Si 2p) at ~ 103 eV, and sulfur (S 2p) at ~ 166 eV. These peaks confirm the successful synthesis and functionalization of GO with 3-MPTMS molecules, as evidenced by the presence of silicon and sulfur signals from the organosilane coupling agent. The intensity and position of these peaks align with previously reported studies on 3-MPTMS functionalized graphene oxide^30^.

Following exposure to Pb²⁺ ions, the XPS survey spectrum of 3-MPTMS-GO + Pb²⁺ exhibits significant modifications. Notably, prominent peaks corresponding to lead appear in the spectrum, with the Pb 4f peak at approximately 140 eV and the Pb 4 d peak at ~ 415 eV. These newly emerged signals demonstrate successful adsorption of lead ions onto the sensor surface. Additionally, the relative intensities of the C 1 s, O 1 s, Si 2p, and S 2p peaks show slight modifications following Pb²⁺ exposure, suggesting that the lead ions interact with the multiple functional groups present on the 3-MPTMS-GO surface, including thiol (-SH), hydroxyl (-OH), carboxyl (-COOH), and silanol (-Si-OH) groups. comprehensive peak fitting for all high-resolution spectra was used to further investigate the behavior and the mechanism of the adsorption of Pb^2+^ on the 3-MPTMS-GO sensor. All peak fitting parameters including binding energies, FWHM values, area percentages, and chemical assignments for all components across all high-resolution spectra are summarized in Table 6 S.

**Fig. 12:**
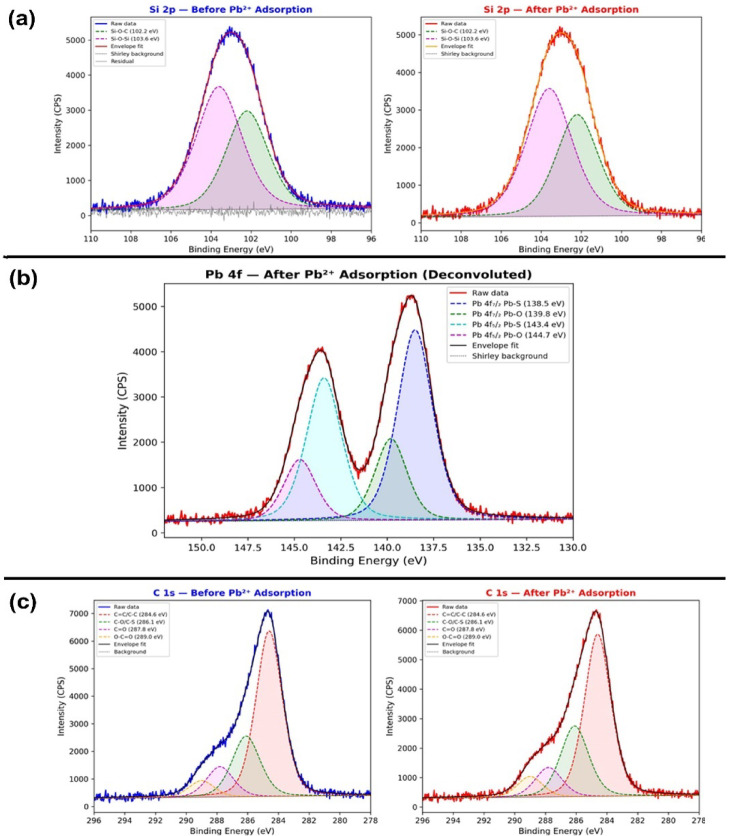
High-resolution XPS spectra for: (**a**) Si 2p of 3-MPTMS-GO before (left, blue) and after (right, red) Pb^2 +^ adsorption, (**b**) Pb 4f of 3-MPTMS-GO after Pb^2 +^ adsorption, (**c**) C 1 s of 3-MPTMS-GO before (left) and after (right) Pb^2 +^ adsorption with Pseudo-Voigt peak deconvolution and Shirley background subtraction.

#### High-resolution of Si 2p spectra analysis

As displayed in Fig. 12a the Si 2p spectrum, previously shown as a single peak, has been deconvoluted into two components: Si-O-C at 102.2 eV (44.4% area) and Si-O-Si at 103.6 eV (55.6% area), consistent with the siloxane network formed during 3-MPTMS functionalization^71^. After Pb^2 +^ adsorption, the peak positions and relative areas remain essentially unchanged, confirming that the silicon-oxygen framework is structurally preserved during lead binding.

#### High-resolution of Pb 4f spectra analysis

As shown in Fig. 12b, the Pb 4f spectrum has been deconvoluted into four components revealing two distinct chemical environments: Pb-S coordination (Pb 4f7/2 at 138.5 eV; Pb 4f5/2 at 143.4 eV) comprising 70% of total Pb signal, and Pb-O coordination (Pb 4f7/2 at 139.8 eV; Pb 4f5/2 at 144.7 eV) comprising 30%^72^. The spin-orbit splitting of 4.9 eV confirms divalent Pb^2+^. The dominant Pb-S component provides direct evidence for thiol-mediated adsorption consistent with HSAB theory^73^, while the Pb-O component confirms secondary binding through oxygen-containing functional groups.

#### High-resolution of C 1 s spectra analysis

The C 1 s spectrum, previously described with only a primary peak and a shoulder, has been deconvoluted into four components: sp2 C = C/C-C at 284.6 eV (49.1%), C-O/C-S at 286.1 eV (28.5%), C = O at 287.8 eV (14.2%), and O-C = O at 289.0 eV (8.2%). After Pb^2 +^ exposure, the sp2 carbon peak decreases slightly while the C-O/C-S component increases as depicted in (Fig. 12c), suggesting modification of the carbon environment through indirect lead coordination with oxygenated carbon species^74^.

**Fig. 13:**
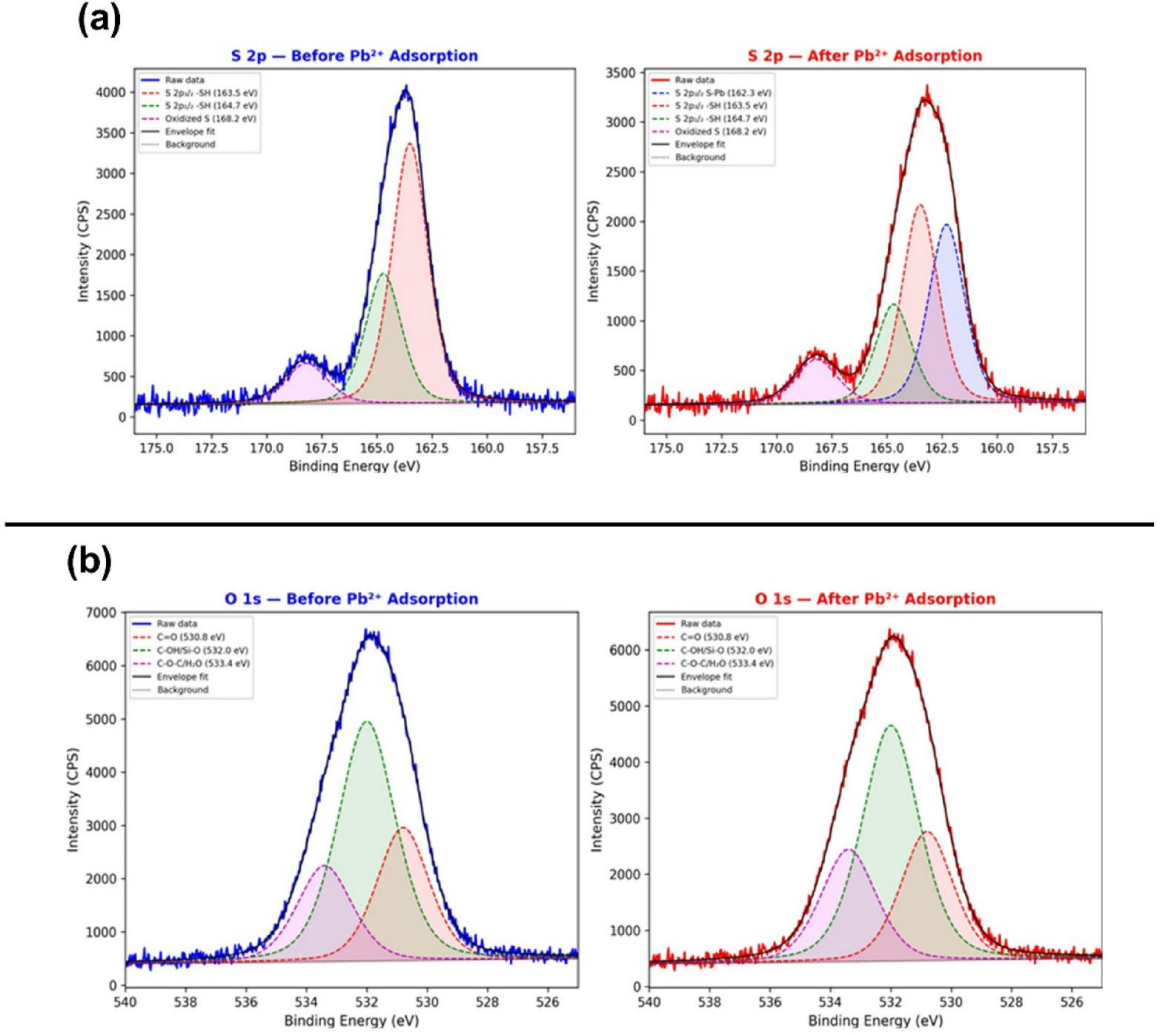
High-resolution XPS spectra for: (**a**) S 2p of 3-MPTMS-GO before (left) and after (right) Pb^2 + ^adsorption, (**b**) O 1 s of 3-MPTMS-GO before (left) and after (right) Pb^2 +^ adsorption.

#### High-resolution of S 2p spectra analysis

The S 2p spectrum before adsorption has been deconvoluted into three components: S 2p3/2 (-SH) at 163.5 eV (53.3%), S 2p1/2 (-SH) at 164.7 eV (26.7%), and oxidized sulfur (SOx) at 168.2 eV (20.0%). After Pb^2 +^ exposure, a critical new component emerges: S-Pb coordination at 162.3 eV (34.3%), while the free -SH components decrease substantially as shown in Fig. 13a. This spectral evolution provides the most direct evidence for thiol-Pb^2 +^ coordination and quantitatively demonstrates that approximately one-third of available thiol groups participate in lead binding^75,76^.

#### High-resolution of O 1 s spectra analysis

As depicted in Fig. 13b, The O 1 s spectrum has been deconvoluted into three components: C = O at 530.8 eV (28.4%), C-OH/Si-O at 532.0 eV (51.1%), and C-O-C/adsorbed H_2_O at 533.4 eV (20.5%). After Pb^2+^ adsorption, subtle changes in relative peaks area confirm secondary involvement of oxygen-containing groups in lead coordination^30^. The C = O component decreases slightly while the C-O-C/H_2_O component increases, consistent with electrostatic interactions between negatively charged oxygen groups and Pb^2 +^ ions^40^.

#### Adsorption mechanism interpretation

Quantitative analysis of the XPS data derived from the survey and high-resolution spectra provides critical insights into the surface composition changes upon Pb²⁺ adsorption. Prior to lead exposure, the atomic percentages on the 3-MPTMS-GO surface were approximately: C (63%), O (28%), Si (5%), and S (4%). Following Pb²⁺ exposure, the surface composition shifted notably to accommodate the newly adsorbed lead, with C (58%), O (25%), Si (4%), S (4%), and Pb (9%). The 9 at% of lead detected on the surface confirms substantial accumulation of Pb²⁺ ions, quantitatively validating the sensor’s adsorption capability. A comprehensive table of the comprehensive XPS analysis collectively demonstrates that the exceptional lead detection performance of the 3-MPTMS-GO-based QCM sensor originates from a multi-functional adsorption mechanism. The dominant primary interaction occurs through thiol-Pb²⁺ coordination, as evidenced by the characteristic Pb 4f doublet at 4.9 eV separation and the S 2p spectral evolution showing S-Pb coordination formation. Secondary interactions involve electrostatic attraction between positively charged Pb²⁺ and negatively charged functional groups (-OH, -COOH, -Si-OH), as confirmed by persistent O 1 s signals and the negative Zeta potential (−31.12 mV) measured for the material. The retention of structural integrity in the graphitic carbon backbone (C 1 s) and silicon framework (Si 2p) during lead binding ensures durability and regeneration capability of the sensor.

These XPS findings provide direct chemical evidence supporting the theoretical basis of this work, where HSAB theory predicts superior affinity between Pb²⁺ (soft acid) and sulfur-containing ligands (soft bases). The presence of multiple binding sites with complementary selectivity for lead ions, combined with the high surface area and roughness of the 3-MPTMS-GO material (as demonstrated by AFM), establishes the foundation for the high sensitivity (LOD = 0.01mg L^-1^), excellent selectivity against competing cations (93–98% signal retention with 4-fold excess interferents), and outstanding regeneration capability (> 94% efficiency after 10 cycles) observed in the QCM sensing experiments.

## Conclusion

An innovative QCM sensor based on 3-MPTMS-GO was successfully designed to detect lead ions (Pb²⁺) in aqueous solutions. Structural and surface characterizations (TEM, Raman, FTIR, XRD, AFM, UV-Vis, contact angle, and Zeta potential) demonstrate the nanomaterial’s successful functionalization, increased hydrophilicity, and improved surface roughness. The inclusion of thiol (-SH), hydroxyl (-OH), and carboxyl (-COOH) groups increased lead ion binding due to significant chemical affinity and electrostatic interactions. The sensor demonstrated a remarkable LOD of 0.01 mg L^-^^1^, which is within WHO-allowable limits. The sensor exhibited excellent selectivity against competing ions and successful performance in real samples. The sensor displayed exceptional regeneration capabilities using EDTA treatment, retaining sensitivity for at least ten cycles and good stability, validating its economic worth. Despite these promising outcomes, the present work represents an early proof-of-concept step. Certain limitations remain, including the relatively short-term stability assessment with need of studies to focus on extended stability testing over months and the need for more extensive real environment estimations and expanding testing to diverse water matrices and real-world environmental conditions. Overall, this work validates that 3-MPTMS-GO is a talented and cost-effective functional material for QCM based heavy metal detection, offering a sensitive, regenerable, and portable platform with strong potential for real-time water-quality monitoring applications.

## Supplementary Information

Below is the link to the electronic supplementary material.

Supplementary Material 1

## Data Availability

The data that support the findings of this study are available from the corresponding author upon reasonable request.
