# Supplementary material for: Evaluation of functionalized graphene oxide as a nanostructured sensor for lead ion detection in aqueous solutions via quartz crystal microbalance
Source: Sci Rep. 2026 May 11;16:14707. doi: 10.1038/s41598-026-50889-1 (PMC13161374; doi:10.1038/s41598-026-50889-1)
Supplement: Supplementary file 1 — Supplementary Material 1 [file 41598_2026_50889_MOESM1_ESM.docx]

**Supplementary Information**

**Table 1S. Calibration data**

| Conc (mg L^-1^) | Rep 1 | Rep 2 | Rep 3 | Mean (Hz) | SD | RSD (%) |
| --- | --- | --- | --- | --- | --- | --- |
| 0.05 | 7.65 | 7.82 | 7.84 | 7.77 | 0.10 | 1.29 |
| 0.07 | 7.72 | 7.85 | 7.86 | 7.81 | 0.08 | 1.02 |
| 0.10 | 7.78 | 7.88 | 7.89 | 7.85 | 0.06 | 0.76 |
| 0.30 | 8.08 | 8.18 | 8.19 | 8.15 | 0.06 | 0.74 |
| 0.50 | 8.35 | 8.48 | 8.46 | 8.43 | 0.07 | 0.83 |
| 0.70 | 8.65 | 8.78 | 8.76 | 8.73 | 0.07 | 0.80 |
| 1.00 | 9.08 | 9.18 | 9.19 | 9.15 | 0.06 | 0.66 |
| 1.50 | 9.82 | 9.95 | 9.93 | 9.90 | 0.07 | 0.71 |

**Table 2S. sensor to sensor reproducibility**

| Sensor ID | @0.1 mg L^-1^ (Hz) | @0.5 mg L^-1^ (Hz) | @1.0 mg L^-1^ (Hz) | LOD (mg L^-1^) |
| --- | --- | --- | --- | --- |
| Sensor 1 | -7.85 | -8.39 | -9.28 | 0.010 |
| Sensor 2 | -7.78 | -8.28 | -9.15 | 0.012 |
| Sensor 3 | -7.92 | -8.45 | -9.35 | 0.009 |
| Sensor 4 | -7.82 | -8.35 | -9.22 | 0.011 |
| Sensor 5 | -7.80 | -8.32 | -9.18 | 0.011 |
| Mean | **-7.83** | **-8.36** | **-9.24** | **0.011** |
| RSD (%) | **0.64** | **0.84** | **0.87** | **9.1** |

**Table 3S. Mixed-Ion Interference Results**

| Test  Condition | Pb^2+^ Conc.  (mg L^-1^) | Interfering Ion  (mg L^-1^) | Response  (Hz) | Recovery  (%) |
| --- | --- | --- | --- | --- |
| Pb^2+^ Only | 0.5 | - | −8.39 ± 0.20 | 100 |
| Pb^2+^ + Mg^2+^ | 0.5 | 2.0 | −8.25 ± 0.30 | 98.3 |
| Pb^2+^ + Cr^3+^ | 0.5 | 2.0 | −8.20 ± 0.25 | 97.7 |
| Pb^2+^ + Ni^2+^ | 0.5 | 2.0 | −8.15 ± 0.20 | 97.1 |
| Pb^2+^ + Ca^2+^ | 0.5 | 2.0 | −8.00 ± 0.10 | 95.3 |
| Pb^2+^ + Fe^3+^ | 0.5 | 2.0 | −7.96 ± 0.80 | 94.8 |
| Pb^2+^ + Cu^2+^ | 0.5 | 2.0 | −7.90 ± 0.84 | 94.1 |
| Pb^2+^ + Cd^2+^ | 0.5 | 2.0 | −7.86 ± 0.40 | 93.6 |
| Pb^2+^ + Zn^2+^ | 0.5 | 2.0 | −7.80 ± 0.60 | 92.9 |

**Table 4S. Multi-Level Interference Analysis**

| Pb²⁺ (mg L^-1^) | Condition | Response (Hz) | Recovery (%) |
| --- | --- | --- | --- |
| 0.01 | Pb²⁺ only | -7.70 ± 0.15 | 100 |
| 0.01 | Mixed ions | -7.12 ± 0.28 | 92.5 |
| 1.00 | Pb²⁺ only | -9.15 ± 0.20 | 100 |
| 1.00 | Mixed ions | -8.32 ± 0.30 | 90.9 |
| 2.00 | Pb²⁺ only | -7.08 ± 0.15 | 100 |
| 2.00 | Mixed ions | -6.55 ± 0.25 | 92.5 |

**Table 5S. Sample Validation Results**

| Sample Type | Baseline Pb²⁺ (mg L^-1^) | QCM Response (Hz) | pH | TDS  (mg L^-1^) | Recovery (%) |
| --- | --- | --- | --- | --- | --- |
| Tap Water | 0.003-0.007 | −2.40 ± 0.3 | 7.1-7.3 | 285-312 | 99.8-103.2 |
| Groundwater | 0.007-0.015 | −6.45 ± 0.4 | 6.8-7.2 | 425-512 | 96.8-98.2 |
| Industrial | 0.05-1.20 | −12.8 ± 0.6 | 5.8-6.5 | 1250-2100 | 91.5-93.1 |

**
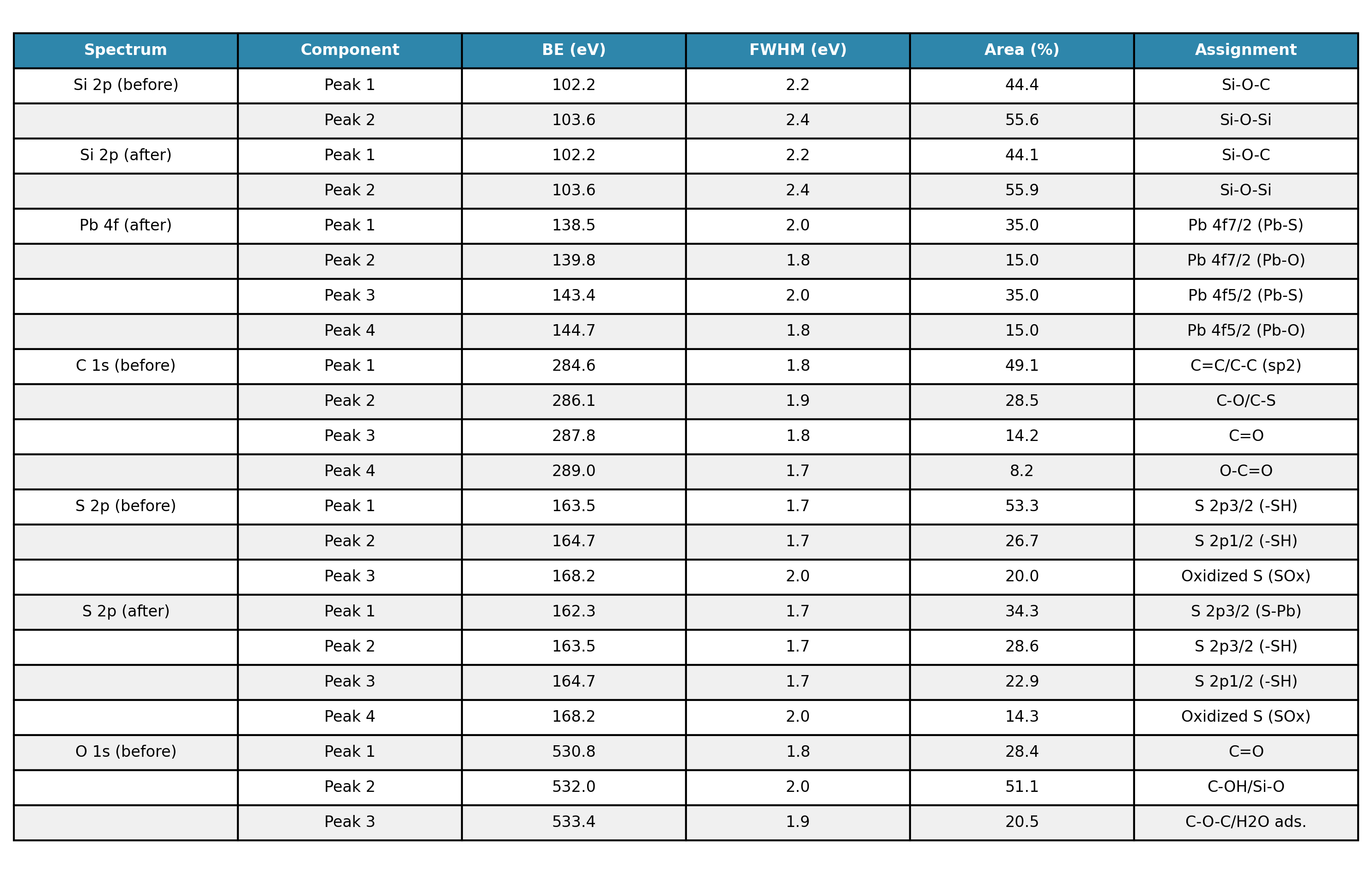
Table 6S. XPS peaks deconvolution parameters (pseudo- Voigt fitting with Shirley background)**

**
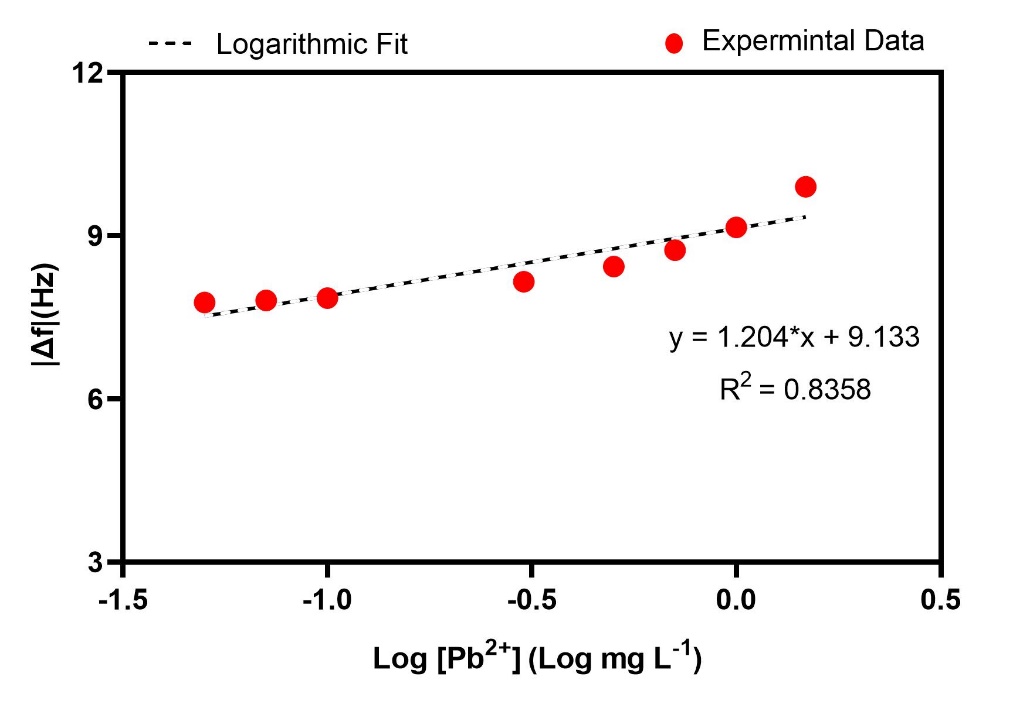
**

**Fig. 1S**. **Detection of Pb²⁺ across (0.05-1.5 mg L^1-^) concentration range by** **the 3-MPTMS-GO-based QCM sensor, Semi-logarithmic plot**


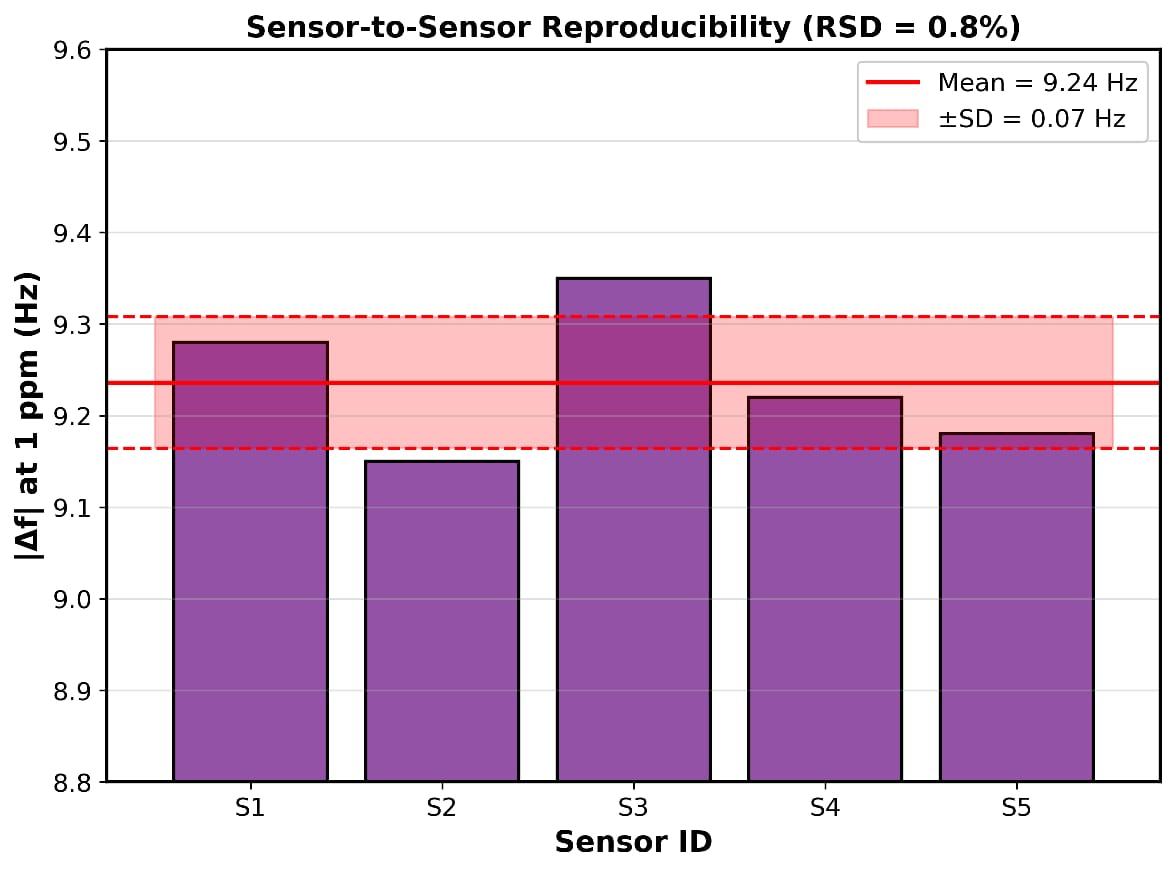


**Fig. 2S. Sensor-to- Sensor Reproducibility for the 3-MPTMS-GO-based QCM sensor**


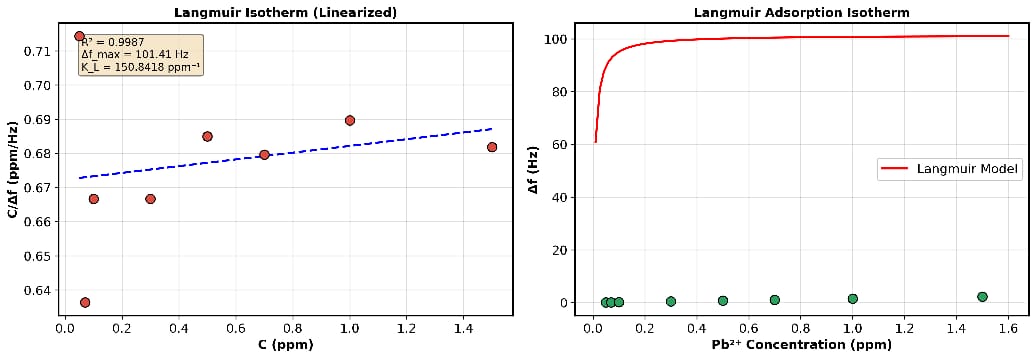


**Fig. 3S. Adsorption isotherm of Pb^2+^ on 3-MPTMS-GO-based QCM sensor, Langmuir model**


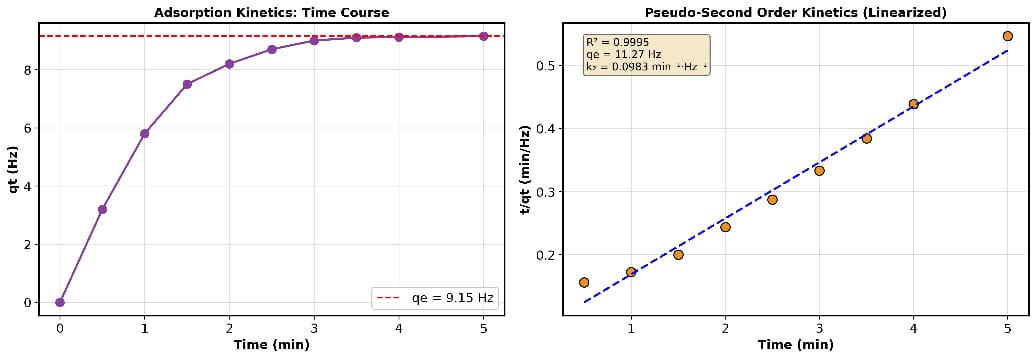


**Fig. 4S. Adsorption kinetics model of Pb^2+^ on 3-MPTMS-GO-based QCM sensor, pseudo-second order** **model**


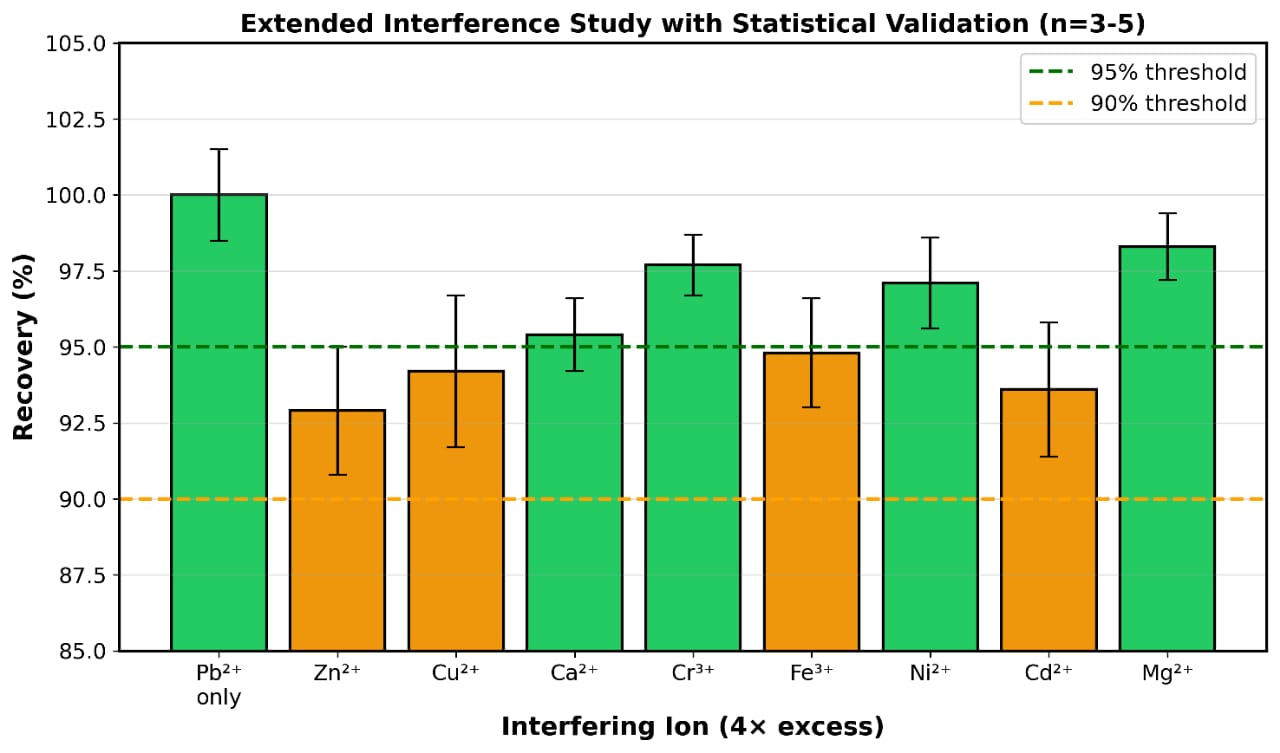
**Fig. 5S. Signal recovery comparison for Pb^2+^ detection in presence of interference ions by 3-MPTMS-GO-based QCM sensor**


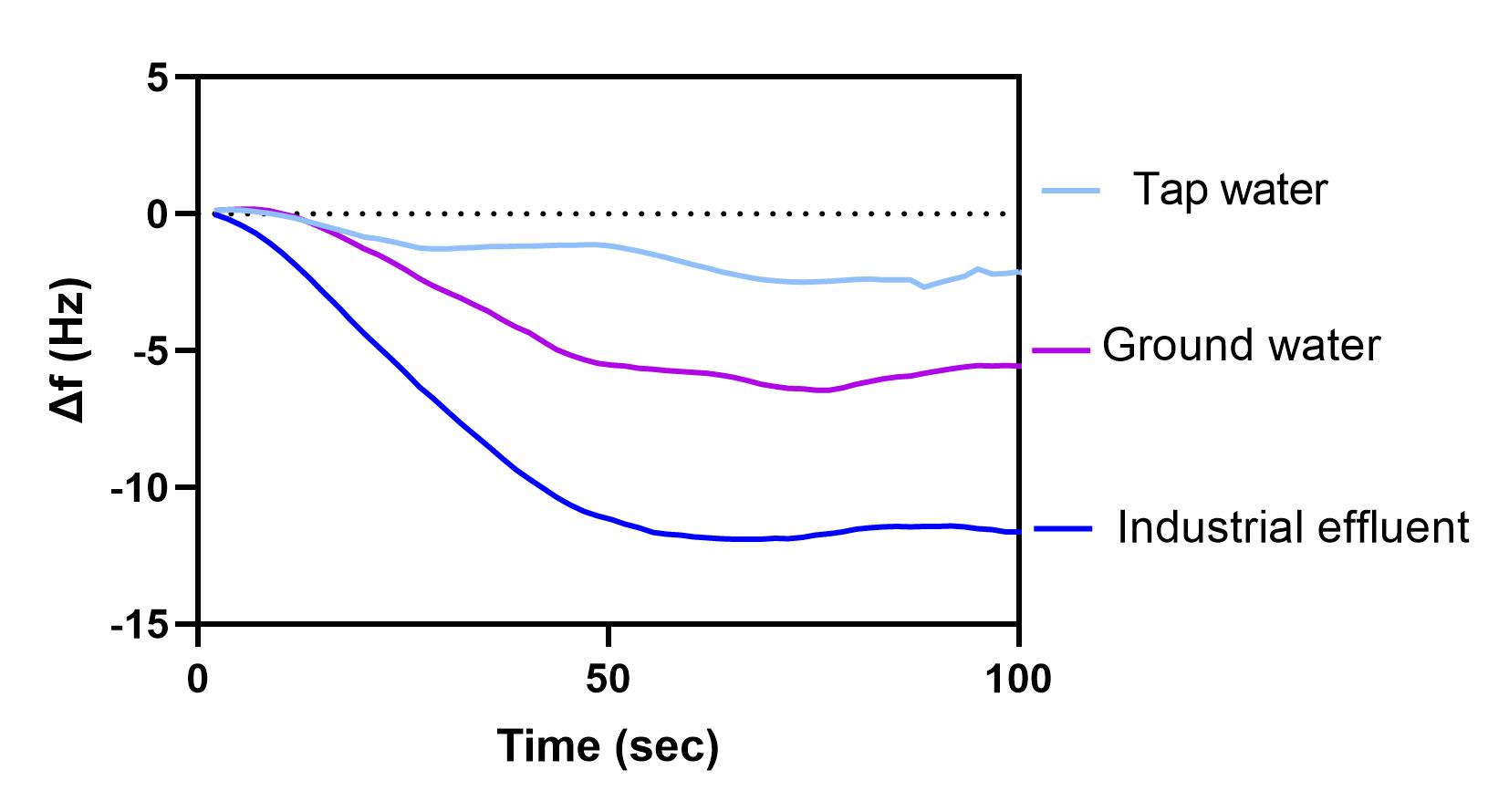


**Fig. 6S. Real-time QCM frequency change (Δf) for real samples measured by** **the 3-MPTMS-GO sensor**


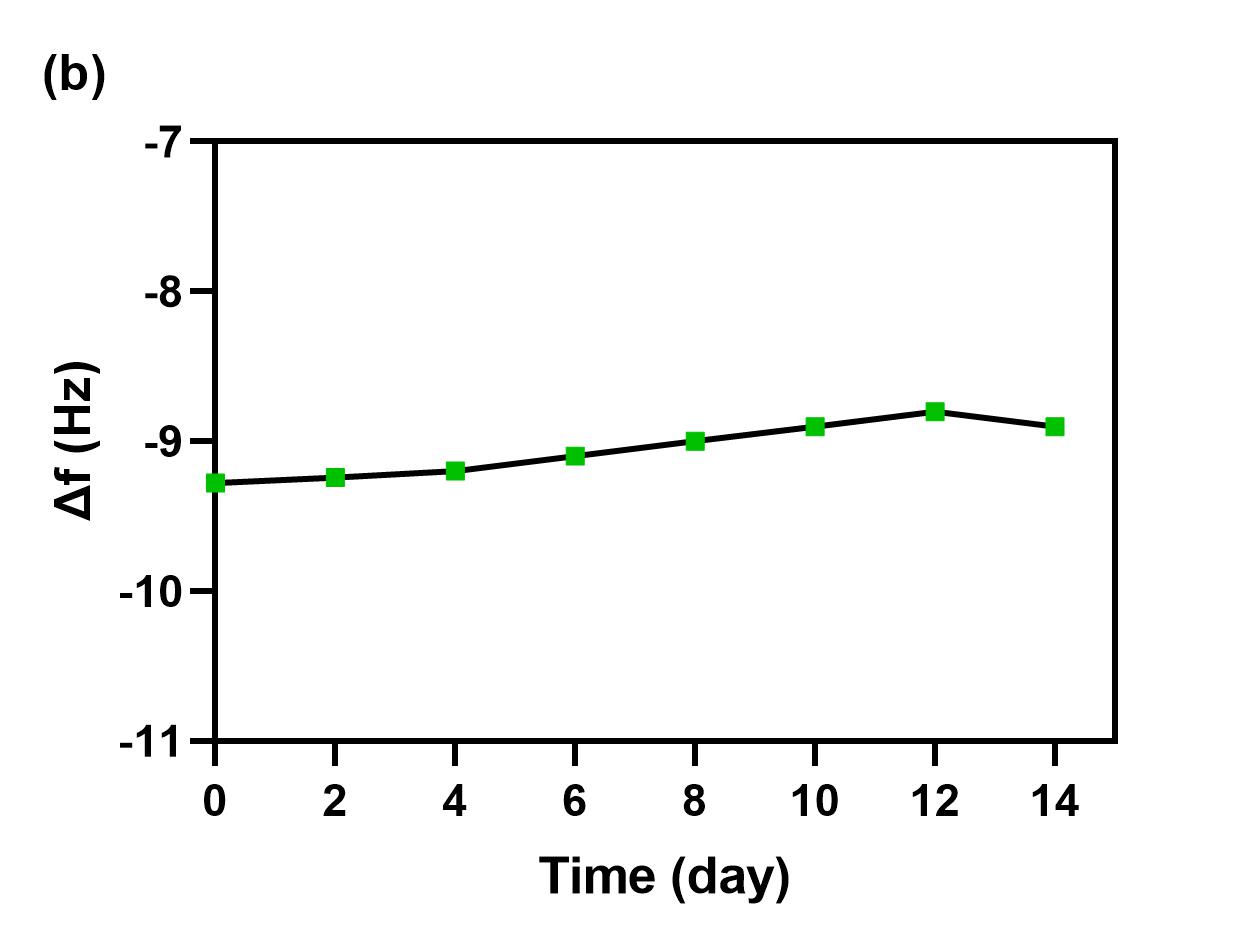

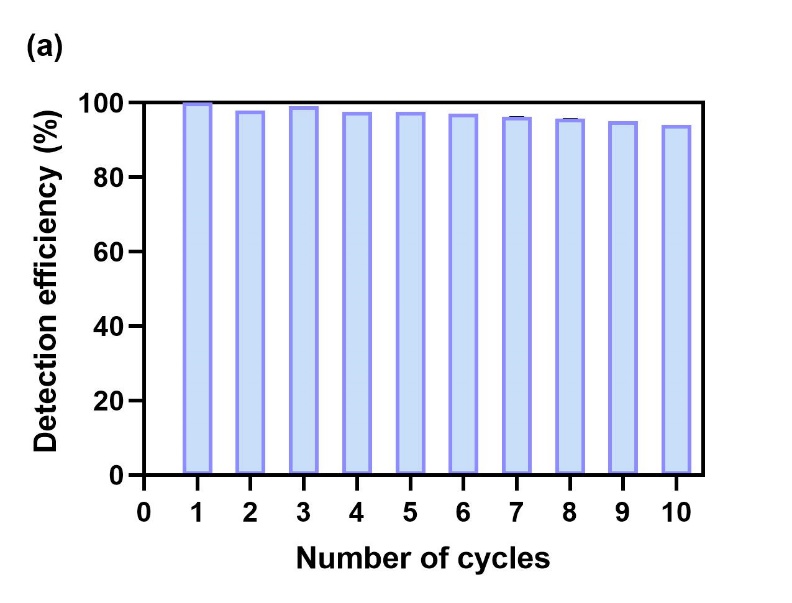
**Fig. 7S. (a) Detection of Pb²⁺ (1 mg L^-1^)** **using the 3-MPTMS-GO sensor within 10 consecutive cycles at neutral** **pH. (b) Frequency response to Pb^2+^ (1 mg L^-1^) recorded by the 3-MPTMS-GO sensor as function of time at neutral pH**
